# pH‐Responsive Nanoplatform‐Mediated Lipid Hijacking and Metabolic Collapse for Synergistic Starvation‐Immunotherapy

**DOI:** 10.1002/advs.78459

**Published:** 2026-10-28

**Authors:** Xianlong Zong, Haochen Yao, Danyang Shen, Furu Gong, Zixuan Wang, Haoyang Qin, Yang Yang, Ruirui Qiao, Yongjuan Li, Rui Li

**Affiliations:** ^1^ Department of Stomatology The First Affiliated Hospital of Zhengzhou University Zhengzhou University Zhengzhou Henan China; ^2^ Tianjian Laboratory of Advanced Biomedical Sciences Academy of Medical Sciences Zhengzhou University Zhengzhou Henan China; ^3^ Australian Institute for Bioengineering and Nanotechnology The University of Queensland QLD Australia; ^4^ Key Laboratory and Engineering Laboratory of Lymphatic Surgery Jilin Province China‐Japan Union Hospital of Jilin University Changchun Jilin China

**Keywords:** cancer‐associated fibroblasts (CAFs), connexin‐43 (Cx43), lipid droplets (LDs), metabolic compensation, starvation‐immunotherapy

## Abstract

Tumor starvation therapy is a promising metabolism‐targeting intervention, yet its efficacy is largely counteracted by lipid droplet (LD)‐driven compensatory metabolism in malignant cells. Existing LDs‐targeted approaches are further hampered by off‐target systemic metabolic disturbances and inherent immunosuppressive adverse effects. Here, we construct a zeolitic imidazolate framework‐8 (ZIF‐8)‐based nanomedicine loaded with 2‐deoxy‐D‐glucose (2‐DG) and surface‐decorated with copper‐tannic acid (Cu‐TA, ZIF‐8/2‐DG@Cu‐TA). This nanosystem reroutes intracellular LDs from tumor cells to adjacent cancer‐associated fibroblasts (CAFs) to realize synergistic tumor starvation and immunotherapy. In the acidic tumor microenvironment, the degradable Cu‐TA coating activates connexin 43 (Cx43) hemichannels on CAFs. After internalization by tumor cells, ZIF‐8/2‐DG releases 2‐DG to suppress glycolysis, while dissociated zinc ions disrupt mitochondrial bioenergetics and further trigger Cx43 hemichannels activation on tumor cells. Fully assembled Cx43 gap junctions mediate directional LDs trafficking from tumor cells to CAFs, restricting intracellular lipid energy pools sustaining malignant metabolic demands. Simultaneous blockade of glycolysis, mitochondrial respiration and lipid metabolism induces profound tumor‐cell metabolic collapse. The resulting metabolic stress strongly amplifies antitumor immunity and markedly suppresses both subcutaneous and orthotopic MOC1 tumors. Overall, this study establishes a gap‐junction‐driven triple metabolic starvation paradigm and offers mechanistic insights for next‐generation metabolic immunotherapies.

## Graphical abstract

A pH‐responsive ZIF‐8/2‐DG@Cu‐TA nanoplatform orchestrates multifaceted metabolic disruption for synergistic starvation‐immunotherapy. Acid‐triggered 2‐DG release blocks glycolysis, while Zn^2+^ overload induces mitochondrial dysfunction. Concurrently, Cx43‐mediated gap junctions redirect lipid droplets from tumor cells to cancer‐associated fibroblasts. This coordinated energy deprivation triggers metabolic collapse, enhances antitumor immunity, and effectively suppresses oral cancer progression.

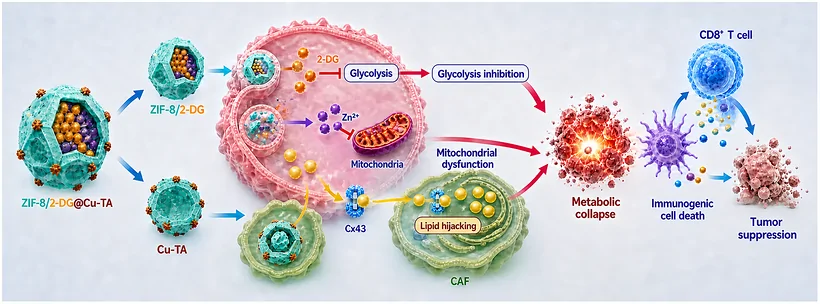

## 1 Introduction

Metabolic tumor‐starvation approaches represent an appealing therapeutic alternative for cancer therapy. They restrain malignant outgrowth by interfering with nutrient delivery and energy‐producing pathways essential for neoplastic cell maintenance and proliferation [1, 2]. Distinct from standard cytotoxic chemotherapies, starvation therapeutics exploit tumor‐intrinsic metabolic flexibility, which may alleviate key drawbacks such as drug resistance and non‐specific toxicity while yielding superior treatment selectivity [3, 4]. However, tumor‐starvation strategies still suffer from considerable practical limitations. Under nutrient‐deprived conditions, tumor cells mount robust adaptive metabolic responses, wherein intracellular lipid droplets (LDs) serve as critical energy‐storage organelles. Once activated, LD catabolism drives alternative metabolic circuits to support neoplastic cell survival, invasiveness and metastatic potential [5]. Such lipid‐driven metabolic reprogramming greatly blunts the therapeutic output of single‐mode starvation interventions, frequently leading to incomplete tumor suppression and eventual treatment failure [6].

Nevertheless, while conventional lipid‑targeted antitumor agents designed to counter this compensatory metabolism act by suppressing lipid biosynthesis, accelerating lipolytic turnover or depleting LDs in tumor cells [7], they still exhibit inherent safety drawbacks [8]. This is because lipid‐metabolic pathways represent evolutionarily conserved physiological machinery across mammalian cells. For small‐molecule lipid‐targeting agents lacking tissue selectivity, off‐target perturbation of such metabolic circuits can disrupt lipid homeostasis within healthy tissues and organs [9]. Moreover, even when such agents are encapsulated within nanocarriers, widespread lipid‐metabolic perturbation in the tumor microenvironment impairs resident immune‐cell activation and function, blunting endogenous antitumor immunity and negating potential metabolic‐immunotherapeutic combinatorial advantages [10]. Thus, developing next‐generation tumor‐starvation therapeutics that simultaneously overcome lipid‐driven metabolic compensation as well as off‑target metabolic and immune toxicities remains a major unmet need, and modulating intercellular metabolite exchange between tumor and stromal cells offers a promising strategy to reshape lipid distribution without broad‐scale metabolic suppression.

Gap junctions are intercellular channels composed of connexin proteins that enable substance exchange between adjacent cells [11]. As the major connexin component of gap junctions in tumors, Cx43 has attracted increasing attention for its pathological roles [12]. In oral squamous cell carcinoma (OSCC), Cx43 frequently displays aberrant expression and impaired channel activity, alterations that are closely linked to malignant progression, metabolic adaptation, and acquired therapeutic resistance [13]. Within this pathological context, CAFs, the predominant stromal component in OSCC lesions, exert critical functions in tumor‐stromal interplay. Existing studies have largely focused on CAFs‐derived metabolic substrates fueling tumor progression [14]. Conversely, CAFs may also acquire tumor‐derived substances to restrain malignant phenotypes or remodel their own functional profiles [15]. Heterocellular Cx43‐dependent communication can be established between CAFs and OSCC cells, offering a direct physical route for intercellular material exchange. On this basis, it is unclear whether excess lipids accumulated in tumor cells could be directionally shuttled to CAFs via this communication axis to achieve “off‑site lipid storage”.

Herein, a ZIF‐8‐based nanomedicine is constructed by encapsulating glycolysis‑targeting 2‐deoxy‐D‐glucose (2‐DG), followed by surface functionalization with copper‑tannic acid (Cu‐TA, ZIF‐8/2‐DG@Cu‐TA). Rather than directly depleting intracellular LDs, ZIF‐8/2‑DG@Cu‐TA manipulates tumor‐CAFs metabolic crosstalk to redirect LDs storage from malignant cells toward CAFs, achieving biosafe synergistic starvation‐immunotherapy (Scheme 1). Mechanistically, Cu‑TA coating undergoes selective degradation within the acidic tumor microenvironment to trigger Cx43 hemichannel opening on CAFs. Upon internalization by tumor cells, ZIF‑8/2‑DG releases 2‑DG to suppress glycolysis and Zn^2^^+^ to disrupt mitochondrial bioenergetics while further promoting Cx43 hemichannel opening on the tumor cells. Assembly of competent Cx43 gap‐junction channels enables concentration‐gradient‐driven LDs transfer and retention within CAFs, which deprives tumor cells of their lipid‑derived energy reserves. Concurrent blockade of glycolysis, mitochondrial respiration, and lipid metabolism induces comprehensive metabolic collapse in tumor cells and elicits robust antitumor immune activation, effectively inhibiting the progression of subcutaneous and orthotopic OSCC. Overall, this work puts forward a triple metabolic‐starvation paradigm relying on stroma‐tumor metabolic crosstalk, which provides novel insights into potent metabolic immunotherapy.

**SCHEME 1:**
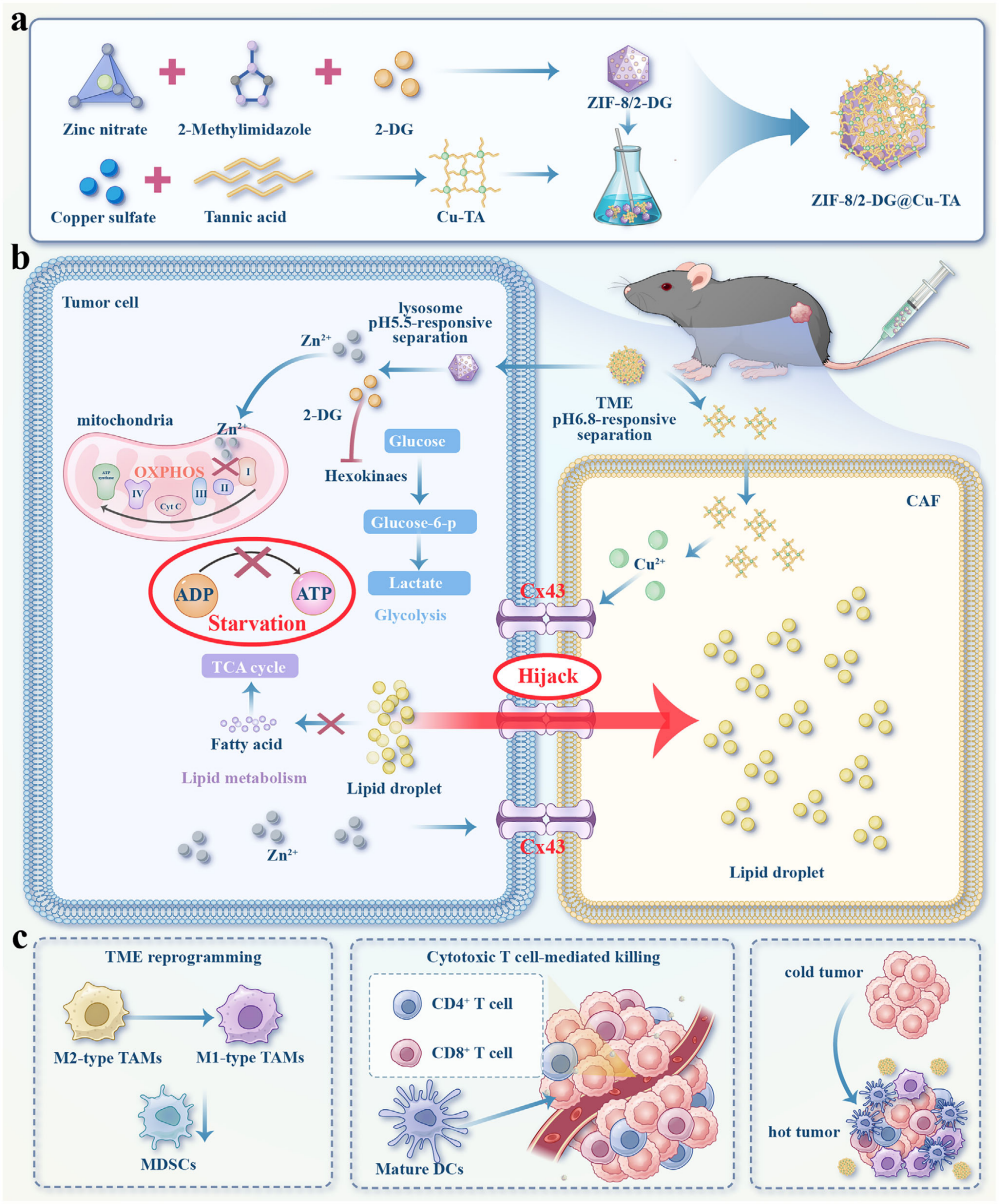
Cx43‑mediated intercellular LDs rerouting to CAFs by ZIF‑8/2‑DG@Cu‑TA for combined tumor starvation‐immunotherapy. (a) Preparation of the ZIF‐8/2‐DG@Cu‐TA nanomedicine. (b) ZIF‐8/2‐DG@Cu‐TA induces glycolysis, mitochondria, and lipid metabolism collapse. (c) ZIF‐8/2‐DG@Cu‐TA reprograms the TME, activates cytotoxic T cell‐mediated killing and transforms “cold” tumor into “hot” ones.

## 2 Results and Discussion

### 2.1 Design and Preparation of the ZIF‐8/2‐DG@Cu‐TA

First, zeolitic imidazolate framework‐8 (ZIF‐8) was constructed as a sacrificial template via aqueous coordination of 2‐methylimidazole and zinc ions (Zn^2+^). From transmission electron microscopy (TEM) images shown in Figure 1a, the synthesized ZIF‐8 exhibited a well‐defined rhombic dodecahedral morphology, which is characteristic of its canonical framework structure. The particles displayed a narrow size distribution centered at ∼70 nm, with favorable dispersity and negligible agglomeration. High‐angle annular dark‐field scanning transmission electron microscopy (HAADF‐STEM) imaging demonstrated that ZIF‐8 had a well‐defined rhombic dodecahedral geometry with an average diameter of 71 nm. Elemental mapping further confirmed the homogeneous distribution of C, N, and Zn across the framework, consistent with the intrinsic chemical composition of ZIF‐8 (Figure 1b). Subsequently, the glycolytic inhibitor 2‐deoxy‐d‐glucose (2‐DG) was encapsulated within ZIF‐8 cavities via physical adsorption, yielding ZIF‐8/2‐DG. Copper ions (Cu^2+^) and tannic acid (TA) were then incorporated into the ZIF‐8/2‐DG dispersion to construct the final ZIF‐8/2‐DG@Cu‐TA nanomedicine. As demonstrated in Figure 1a, ZIF‐8/2‐DG@Cu‐TA exhibited a spherical‐like coated morphology, with a rougher surface attributed to the Cu‐TA layer modification. Elemental mapping of ZIF‐8/2‐DG@Cu‐TA clearly visualized the presence of O and Cu (in addition to the inherent C, N, Zn of ZIF‐8) (Figure 1c), while energy‐dispersive X‐ray spectroscopy (EDS) further confirmed the elemental composition consistency with the designed structure (Figure 1d). X‐ray photoelectron spectroscopy (XPS) was utilized to characterize the chemical states and interfacial bonding of the nanomedicine (Figure S1). Zn 2p signals located at 1021.6 eV (Zn 2p_3_/_2_) and 1044.7 eV (Zn 2p_1_/_2_) verified the preservation of Zn‑N coordination within the ZIF‑8 core (Figure 1e and Figure S2). The Cu 2p spectrum displayed major peaks at 934.6 eV (Cu 2p_3_/_2_), 954.4 eV (Cu 2p_1_/_2_), alongside satellite features near 942 and 962 eV, confirming Cu^2^^+^‑TA chelation and metal‑polyphenol network formation (Figure 1f and Figure S3). The O 1s component centered at 532.8 eV was assigned to C‐O‐H moieties originating from 2‑DG (Figure S4). Using NBD‐tagged 2‐deoxy‐D‐glucose (2‐NBDG) as a fluorescent surrogate, we calculated the 2‑DG loading content to be 20.2% (Figure S5). As determined by dynamic light scattering (DLS), ZIF‐8/2‐DG@Cu‐TA had an average hydrodynamic diameter of 217.7 nm with a low polydispersity index (PDI = 0.11, Figure 1g). Cu‑TA shell deposition reversed the surface zeta potential from +27.9 mV for ZIF‑8/2‑DG to ‐35.2 mV (Figure S6). This reversed surface charge feature helps mitigate undesired protein adsorption in blood and confers superior colloidal stability.

**FIGURE 1:**
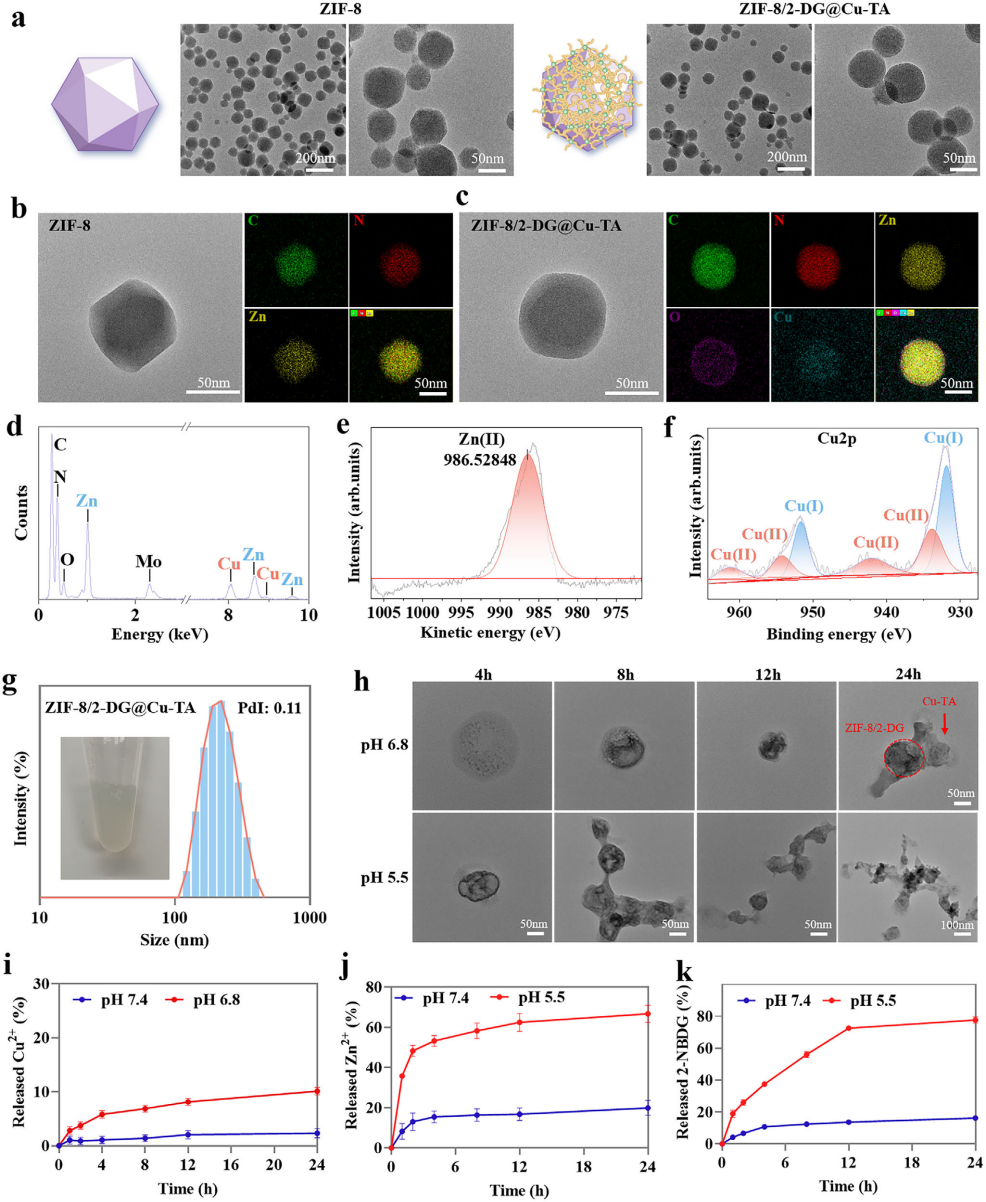
Preparation and characterization of ZIF‐8/2‐DG@Cu‐TA. (a) TEM images of ZIF‐8 and ZIF‐8/2‐DG@Cu‐TA. Elemental mapping of (b) ZIF‐8 and (c) ZIF‐8/2‐DG@Cu‐TA. (d) EDS spectra of ZIF‐8/2‐DG@Cu‐TA. (e,f) XPS spectra of ZIF‐8/2‐DG@Cu‐TA. (g) Size distribution of ZIF‐8/2‐DG@Cu‐TA. (h) TEM images of ZIF‐8@Cu‐TA in different pH solutions. (i–k) Cu^2+^, Zn^2+^, and 2‐DG release profiles of ZIF‐8/2‐DG@Cu‐TA in different pH solutions. Data are expressed as means ± SD (*n* = 3).

Tumor tissues establish an acidic microenvironment (pH 6.5‐6.8) due to elevated aerobic glycolysis [16], which triggers electrostatic repulsion and partial detachment of the Cu‐TA coating [17]. In addition, ZIF‐8‐based nanoparticles were internalized by tumor cells via endocytosis and underwent full disintegration in highly acidic lysosomes [18]. TEM imaging and Tyndall effect were then studied at pH 6.8 and 5.5 to simulate the tumor microenvironment and lysosomal compartment, respectively. As shown in Figure 1h, ZIF‐8@Cu‐TA underwent core‐shell dissociation at pH 6.8, whereas its overall particle morphology was preserved. The sustained yet attenuated Tyndall effect points to partial disassembly and aggregation of the outer Cu‑TA network, even as the ZIF‑8 core maintains structural integrity. We further incubated ZIF‐8@Cu‐TA in pH 5.5 buffer. Under this condition, the spherical nanoparticle architecture was fully disrupted, and the Tyndall effect vanished (Figure S7), confirming complete degradation of the ZIF‐8 core. These gradient‐pH incubation results verified the stepwise responsive degradation behavior of ZIF‐8@Cu‐TA. Finally, we clarified the spatiotemporal release behavior of ZIF‐8/2‐DG@Cu‐TA in Tris buffer at varying pH values (pH 7.4, 6.8, and 5.5). At pH 6.8, the 24 h cumulative Cu^2^^+^ release was 10.1%, driven by acid‑weakened coordination between Cu^2^^+^ and TA phenolic moieties (Figure 1i). Electrostatic repulsion under this pH induced integral‑layer delamination of Cu‑TA network from ZIF‑8, in agreement with the TEM observations. When pH further decreased to 5.5, complete degradation of the ZIF‑8 core drove synchronous release of Zn^2^^+^ and encapsulated 2‑DG. The 24 h release rates of Zn^2^^+^ and 2‑DG were 19.9% and 16.1% at pH 7.4, and increased to 66.7% and 77.6% at pH 5.5, respectively (Figure 1j,k). The slightly higher release of 2‑DG relative to Zn^2^^+^ presumably stemmed from differing diffusion kinetics between small‑molecule 2‑DG and Zn^2^^+^.

### 2.2 Cx43 Activation of CAFs and MOC1 Cells via the ZIF‐8/2‐DG@Cu‐TA

3T3 cells were treated with TGF‑β for 24 h to generate CAFs, as validated by Western blot, PCR, Transwell, and wound‑healing assays (Figure S8) [19, 20]. To probe the cellular localization of ZIF‐8/2‐DG@Cu‐TA, we established a DiO‐labelled CAF/MOC1 co‐culture system as shown in Figure 2a. Fluorescence imaging revealed prominent co‐localization between red Cu^2^^+^ signals and green DiO‑labelled CAFs (Figure 2b), demonstrating preferential Cu‑TA accumulation within CAFs rather than MOC1 cells [21]. This cell‐selective uptake was associated with higher Cu^2^^+^ demand in CAFs and the intrinsic affinity of their abundant scavenger receptors toward polyphenolic moieties [22]. To verify the role of these scavenger receptors, CAFs were treated with CD36 inhibitor Sulfo‐N‐Succinimidyl Oleate Sodium (SSO) and SR‐B1 inhibitor Block lipid transport‐1 (BLT‐1), followed by imaging of intracellular Cu^2^^+^‑derived red fluorescence. Either CD36 or SR‑B1 inhibition attenuated intracellular fluorescence in CAFs, with SSO exerting a stronger effect than BLT‑1; combined inhibitor treatment yielded the weakest signal (Figure S9). Together, these inhibitor‑treatment data point to CD36 and SR‑B1 as key scavenger receptors governing Cu‑TA uptake by CAFs. Subsequently, ZIF‐8/2‐DG core was efficiently internalized by MOC1 cells via endocytosis. Upon lysosomal entry, the acidic lysosomal microenvironment triggered ZIF‑8 framework degradation, enabling sustained cytoplasmic release of Zn^2^^+^ and 2‑DG into MOC1 cells. As shown in Figure 2c, the 2‑NBDG fluorescence shifted rightward with prolonged incubation, indicating time‑dependent cellular internalisation of ZIF‐8/2‐DG. Additional 2‑NBDG fluorescence imaging revealed stronger signals in MOC1 cells treated with ZIF‑8/2‑NBDG and ZIF‑8/2‑NBDG@Cu‑TA relative to free 2‑NBDG, demonstrating improved 2‑NBDG delivery upon nanosystem encapsulation. In addition, MOC1 cells exhibited higher green fluorescence intensity than CAFs, suggesting greater ZIF‑8/2‑NBDG uptake activity in tumor cells (Figure S10), which presumably stemmed from elevated tumor cell metabolic activity. We further assessed intracellular Zn^2^^+^ distribution by fluorescence imaging (Figure 2d). Diffuse blue fluorescence was detected for free Zn^2^^+^ treatment, demonstrating cellular uptake of exogenous Zn^2^^+^, yet its signal intensity was lower than that of the ZIF‑8 and ZIF‑8/2‑DG groups. These imaging observations indicated that ZIF‑8/2‑DG preferentially entered MOC1 cells rather than Zn(NO_3_)_2_.

**FIGURE 2:**
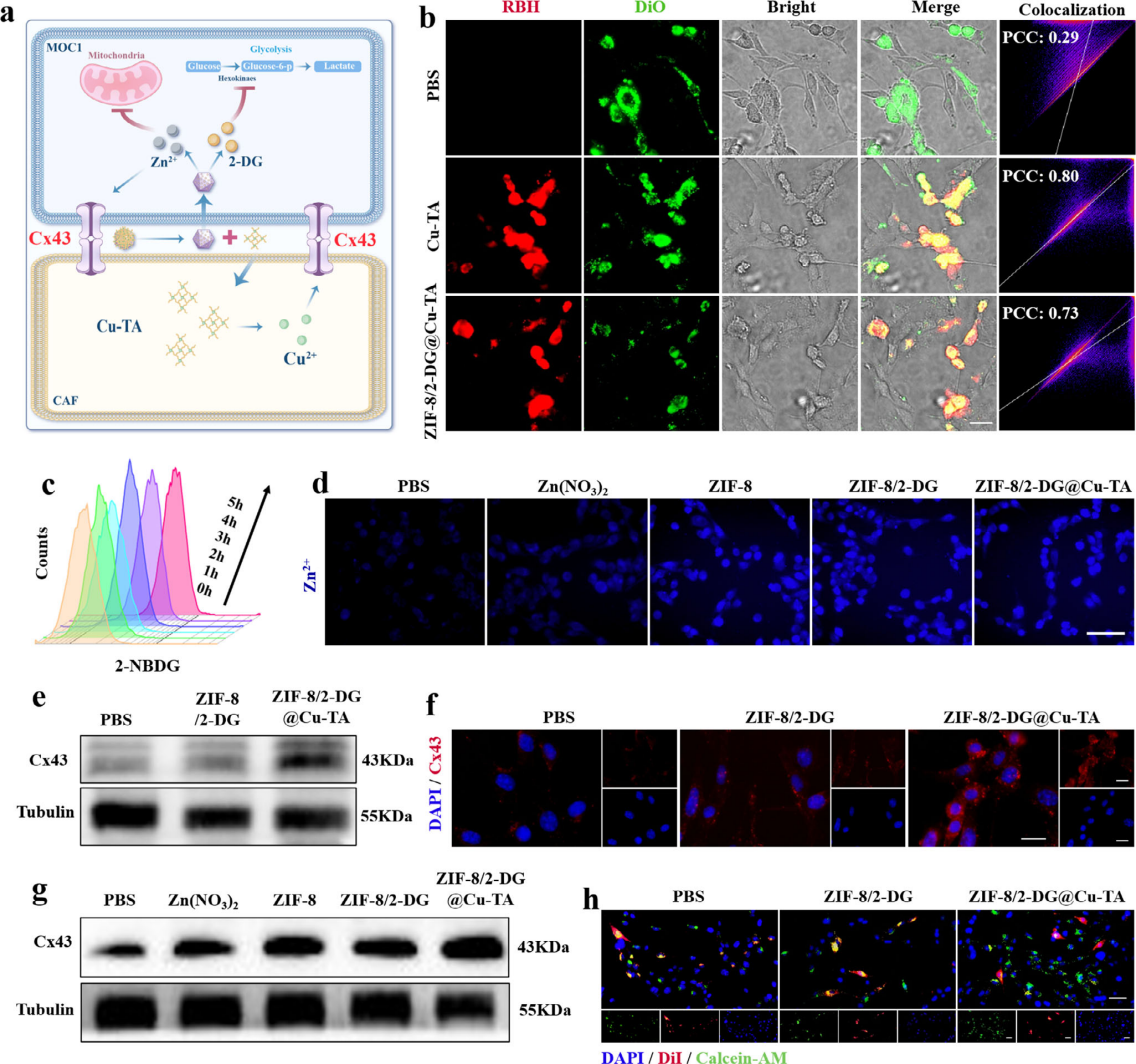
ZIF‐8/2‐DG@Cu‐TA activates Cx43 in CAF and MOC1 cells. (a) Schematic diagram of the degradation behavior and cell‐type‐specific targeting capability of ZIF‐8/2‐DG@Cu‐TA. (b) Fluorescence images of Cu^2+^ in the co‐culture system of DiO‐CAF and MOC1 cells (scale bar: 20 µm). (c) Flow cytometric analysis of 2‐NBDG in MOC1 cells. (d) Fluorescence images of Zn^2+^ in MOC1 cells (scale bar: 50 µm). (e) Western blot analysis of Cx43 in CAFs. (f) Immunofluorescence images of Cx43 in CAFs (scale bar: 20 µm). (g) Western blot analysis of Cx43 in MOC1 cells. (h) Fluorescence images of Calcein‐AM in the CAF/DiI‐MOC1 co‐culture system after treatments (scale bar: 50 µm).

Given the cell‑selective uptake behavior of individual components, we further investigated how ZIF‑8/2‑DG@Cu‐TA treatments modulate functional Cx43 expression in CAFs and MOC1 cells. Western blot assays (Figure 2e and Figure S11a) revealed that ZIF‑8/2‑DG alone failed to up‑regulate Cx43 in TGF‑β‑induced 3T3‑derived CAFs relative to the PBS group; by contrast, ZIF‑8/2‑DG@Cu‑TA markedly elevated Cx43 abundance in CAFs, as also supported by immunofluorescence observations (Figure 2f). To further validate this finding, parallel experiments were performed using human oral cancer‑associated fibroblasts (HOCAFs). Western blot data likewise confirmed Cx43 up‑regulation exclusively upon ZIF‑8/2‑DG@Cu‑TA treatment (Figure S11b). Consistent with preferential CAFs uptake of Cu‑TA, these results suggest that Cu‑TA may dissociate from ZIF‑8 and potentially activate Cx43 hemichannels on the CAFs membrane. This effect can be partially explained by Cu^2^^+^ binding to cysteine residues of Cx43 to modulate its phosphorylation. Cu^2^^+^ suppresses protein phosphatase 2A activity to preserve Cx43 phosphorylation (p‐Cx43) and attenuate its ubiquitin‑dependent degradation [23, 24]. Meanwhile, p‐Cx43 phosphorylation facilitates its membrane translocation and assembly into functional hemichannels [25]. We next quantified Cx43 abundance in MOC1 cells. Cx43 maintained basal expression in the PBS group, while free Zn(NO_3_)_2_ treatment triggered only mild Cx43 elevation. Both ZIF‑8 and ZIF‑8/2‑DG treatments markedly increased Cx43 abundance relative to the PBS group, indicating that the ZIF‑8 nanocarrier can up‑regulate Cx43, presumably via its released Zn^2^^+^. Notably, the ZIF‑8/2‑DG@Cu‑TA group exhibited the highest Cx43 protein level (Figure 2g). We speculated that trace Cu^2^^+^ released from Cu‑TA entered MOC1 cells and acted synergistically with Zn^2^^+^ to up‐regulate Cx43. Mechanistically, Zn^2+^ may bind to the zinc‐finger domain of Cx43, altering its conformation to reduce recognition by ubiquitin ligase, thereby attenuating ubiquitination‐dependent proteasomal degradation and promoting Cx43 accumulation [26]. Collectively, these data reveal cell‑type‑dependent Cx43 modulation triggered by distinct metal‑ion sources within the nanosystem [15].

To validate intercellular molecular transport between CAFs and MOC1 cells in co‑culture, we employed Calcein‑AM. Figure 2h shows markedly elevated perinuclear green fluorescence in CAFs from the ZIF‑8/2‑DG@Cu‑TA group, while the ZIF‑8/2‑DG‑only group displayed faint yet detectable fluorescence with substantially lower signal intensity. This observation may stem from low constitutive Cx43 expression at the cell membrane. Activation of Cx43 hemichannels solely within MOC1 cells fails to sustain efficient intercellular substance exchange. Assembling functional gap‐junction channels necessitates coordinated opening of Cx43 hemichannels on MOC1 cells and nearby CAFs, which facilitates concentration gradient powered dye transfer between adjoining cells. This finding also underpins the rationale for our dual‑cell‑targeting nanomedicine design.

### 2.3 ZIF‐8/2‐DG@Cu‐TA Facilitates Cx43‐Mediated LDs Competition in MOC1 Cells and CAFs

We further explored whether this nanosystem could mediate intercellular LDs translocation, as illustrated in Figure 3a. We hypothesized that Cx43‐based gap junctions enable LDs translocation from MOC1 cells to CAFs. This process depletes the functional LDs pool within MOC1 cells and aggravates their energy exhaustion. Instead of feeding MOC1 cells in a reciprocal manner, the translocated LDs are largely retained by CAFs. This creates competitive metabolic crosstalk that supports antitumor function. In the co‑culture system, PBS‑treated MOC1 cells exhibited high basal LDs abundance, whereas the CAFs showed low intrinsic LDs levels, indicating an LDs concentration gradient between the two cells that provides the driving force for intercellular LDs transfer [27]. Upon ZIF‑8/2‑DG@Cu‑TA treatment, CAFs adjacent to MOC1 cells displayed markedly elevated green fluorescence, suggesting directional LDs transfer from MOC1 to CAFs [28]. In contrast, CAFs adjacent to MOC1 cells in ZIF‑8/2‑DG group exhibited no notable elevation in LDs fluorescence. This phenomenon likely arises from insufficient basal Cx43 expression in CAFs (Figure 3b). To rule out direct nanomedicine‑triggered LDs accumulation in CAFs, monocultured CAFs were treated with ZIF‑8/2‑DG@Cu‑TA. No obvious change in intracellular LDs content was detected (Figure S12). These findings demonstrate that LDs accumulation in CAFs relies on physical proximity to MOC1 cells rather than direct stimulation by the ZIF‑8/2‑DG@Cu‑TA nanomedicine. Given that canonical Cx43 gap‐junction channels only permit diffusion of molecules smaller than 1000 Da [29], an apparent paradox emerges. LDs are complex lipid‑protein organelles without a defined molecular weight and cannot readily pass through conventional channel pores. A plausible mechanistic interpretation may come from a previously reported non‑canonical transport route, in which Cx43 gap‑junction plaques internalize to form double‑membrane connexosome vesicles capable of shuttling intact organelles between neighbouring cells independent of pore‑size constraints [30]. Despite the absence of direct visualization of connexosome structures in our work, vesicle‐associated processes are a plausible explanation for the observed intercellular LDs transfer.

**FIGURE 3:**
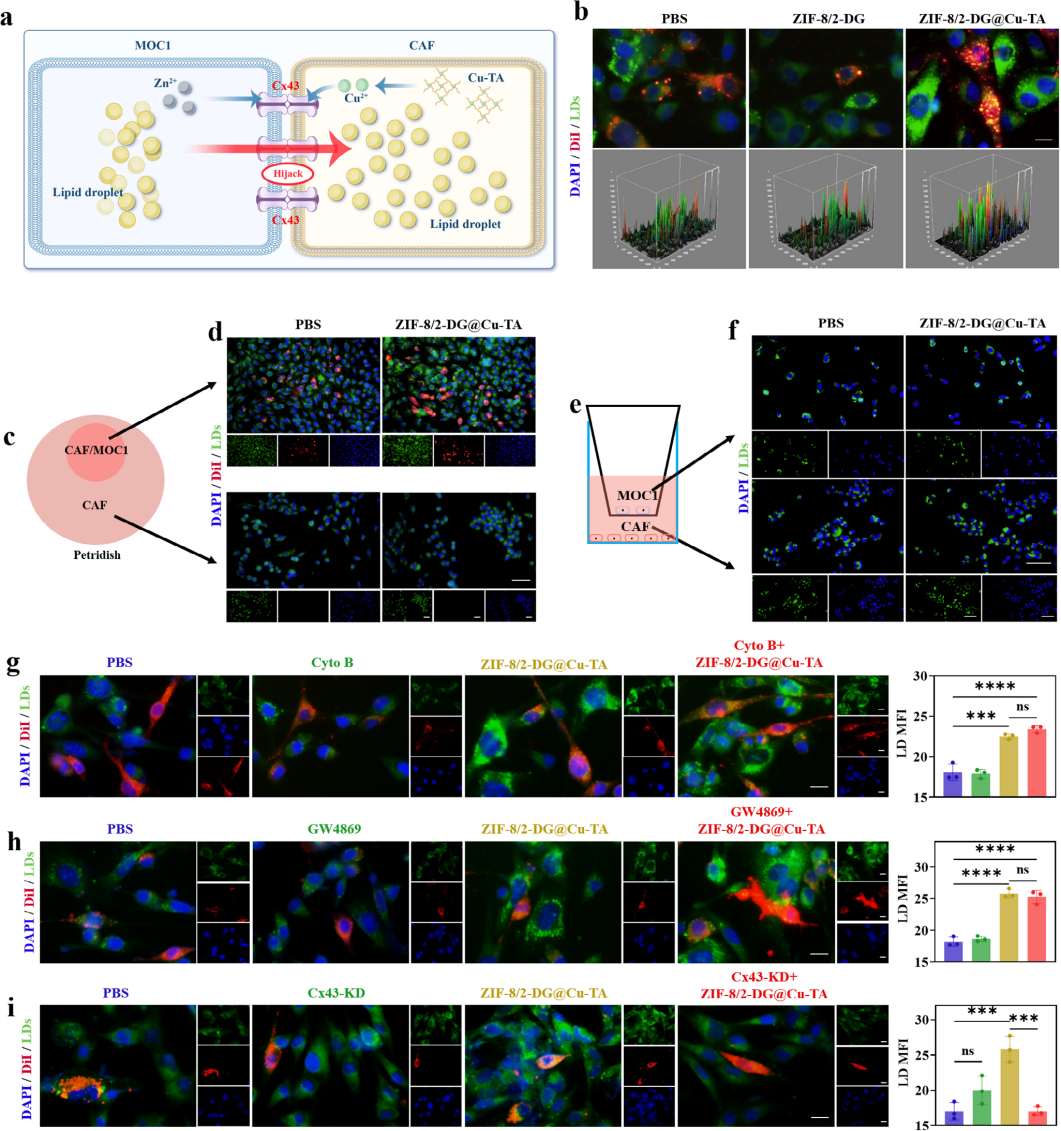
ZIF‑8/2‑DG@Cu‑TA‑enabled Cx43‑mediated gap junctions drive LDs transfer from MOC1 cells to CAFs. (a) Schematic illustration of the predicted mechanism. (b) Fluorescence images of LDs in the CAFs/DiI‐MOC1 co‐culture system (scale bar: 20 µm). (c) Schematic diagram of the partial seeding system. (d) Fluorescence images of LDs in CAFs and DiI‐MOC1 cells after ZIF‐8/2‐DG@Cu‐TA treatment (scale bar: 50 µm). (e) Schematic diagram of the Transwell co‐culture system. (f) Fluorescence images of LDs in CAFs and DiI‐MOC1 cells after ZIF‐8/2‐DG@Cu‐TA treatment (scale bar: 50 µm). (g,h) Fluorescence images and quantitative analysis of LDs in the co‐culture system (scale bar: 20 µm). (i) Fluorescence images and quantitative analysis of LDs in different co‐culture systems following ZIF‐8/2‐DG@Cu‐TA treatment (scale bar: 20 µm). Data are expressed as means ± SD (*n* = 3). Statistical significance was calculated by one‐way ANOVA with Tukey's post hoc test. ^***^ *p* < 0.001, ^****^ *p* < 0.0001 and *p* > 0.05 indicates no significant difference.

To confirm whether this process relies on direct cell‐to‐cell contact, we established a compartmentalized co‑culture system (Figure 3c). As illustrated in Figure 3d, green fluorescence in the PBS group displayed negligible alteration, implying that LDs redistribution does not occur via passive diffusion‐driven mechanisms. Upon ZIF‑8/2‑DG@Cu‑TA treatment, elevated green fluorescence intensities were observed exclusively for CAFs residing in MOC1‑contacting co‑culture compartments, whereas CAFs within isolated compartments retained green fluorescence intensity comparable to control groups. Consistent with earlier observations, these findings suggest that ZIF‑8/2‑DG@Cu‑TA treatment facilitates the assembly of functional gap‑junction channels at cell‑contact sites to promote intercellular LDs transfer. We further physically separated CAFs and MOC1 cells using Transwell inserts (Figure 3e). Under such non‑contact culture conditions, neither the ZIF‑8/2‑DG@Cu‑TA‑treated group nor the PBS control exhibited obvious changes in green fluorescence, confirming that direct cell‑cell contact is indispensable for LDs hijacking (Figure 3f).

Beyond gap junctions, intercellular communication can be mediated by tunneling nanotubes (TNTs) or exosomes [31]. To exclude the involvement of these pathways in LDs translocation, specific inhibitors were supplemented into the co‑culture system, with cytochalasin B to block TNT formation and GW4869 to suppress exosome secretion. As shown in Figure 3g, marked elevation of green fluorescence was observed in CAFs adjacent to MOC1 cells following ZIF‑8/2‑DG@Cu‑TA treatment, yet CytoB‑mediated inhibition failed to abrogate this increase. It thus rules out TNTs as key mediators for intercellular LDs translocation. Consistent results from Figure 3h ruled out the contribution of exosomes to CAF‑mediated LDs sequestration. To further verify the essential requirement of Cx43 for LDs translocation, MOC1 cells with Cx43 knockdown (Cx43‑KD) were generated via RNA interference. Western blot and PCR analyses confirmed prominent down‑regulation of Cx43 at both transcript and protein levels (Figure S13). For functional validation of Cx43 channels, A23187 was applied to open Cx43 channels on MOC1 cells, which triggered robust extracellular ATP release. This ATP efflux was substantially abrogated following Cx43 knockdown, corroborating that Cx43‑formed channels constitute an essential pathway for plasma‑membrane‑associated substance exchange (Figure S13c). We co‑cultured CAFs with either Cx43‑KD MOC1 or MOC1 cells; in the former system, ZIF‑8/2‑DG@Cu‑TA treatment failed to induce LDs accumulation in CAFs (Figure 3i), confirming that functional Cx43 gap junctions are critical for LDs hijacking.

### 2.4 Triple Starvation Therapy With ZIF‐8/2‐DG@Cu‐TA Treatment In Vitro

We measured intracellular ATP levels, the primary cellular energy currency, in MOC1 cells to verify the tumor metabolic‑starvation effect triggered by ZIF‑8/2‑DG@Cu‑TA. 2‑DG suppresses glycolysis by competing with glucose for hexokinase, the rate‑limiting glycolytic enzyme, whereas Zn^2^^+^ impairs mitochondrial function. As shown in Figure 4a, either ZIF‑8/2‑DG or ZIF‑8/2‑DG@Cu‑TA treatment induced a time‑dependent total ATP decrease in MOC1 cells. At 3 h, mitochondrial ATP generation was markedly inhibited in both groups, accompanied by compensatory upregulation of glycolysis‑derived ATP (Figure S14). This observation aligns with the Warburg effect and reflects tumor metabolic plasticity [32]. By 6 h, suppression of mitochondrial ATP was further aggravated, and the compensatory glycolytic response was abrogated through the synergistic action of 2‑DG and Zn^2^^+^, driving cells into a state of complete energy exhaustion. Total ATP dropped to comparably low levels in both groups without obvious differences, attributed to the fact that the Cu‑TA component predominantly dissociates under extracellular acidic conditions, with only a minimal fraction internalized into MOC1 cells (Figure 4b).

**FIGURE 4:**
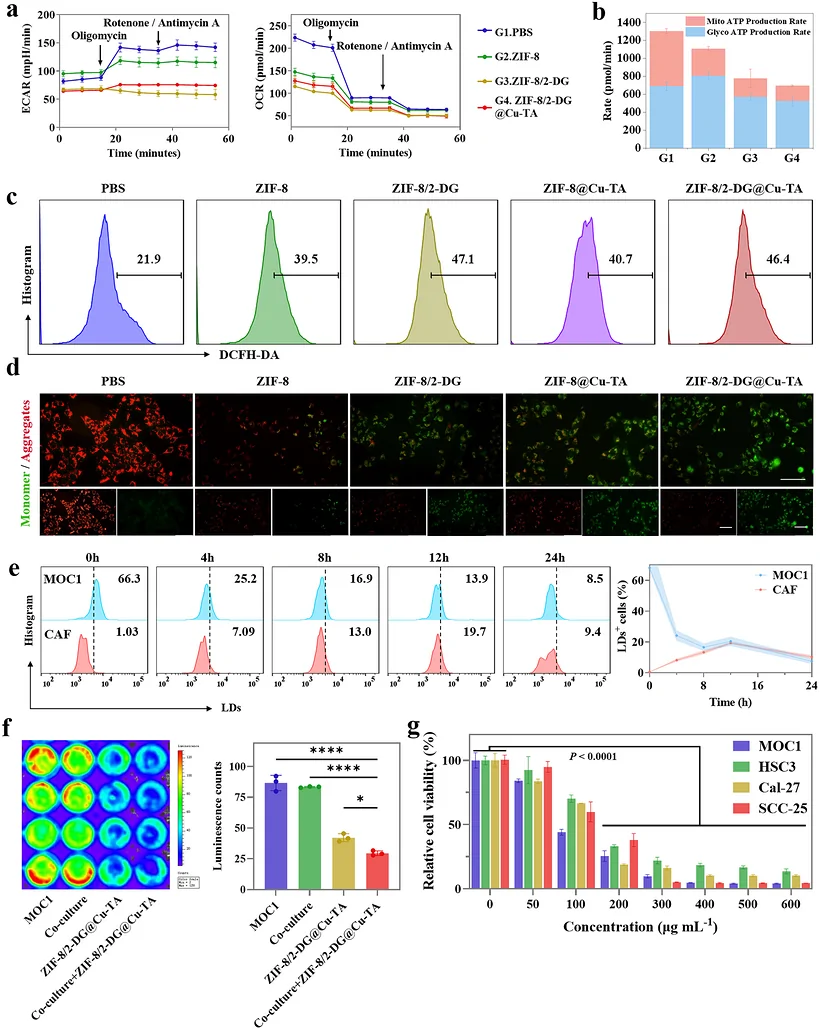
ZIF‐8/2‐DG@Cu‐TA mediates triple starvation therapy in MOC1 cells. (a) ECAR, OCR, and ATP production rates in MOC1 cells treated sequentially with oligomycin, rotenone, and antimycin A. (b) Quantitative analysis of ATP production rate in MOC1 cells. (c) Flow cytometric analysis of intracellular ROS via DCFH‐DA. (d) Fluorescence images of mitochondria in MOC1 cells probed with JC‐1 (scale bar: 50 µm). (e) Flow cytometric analysis of LDs in the MOC1/CAFs co‐culture system after ZIF‐8/2‐DG@Cu‐TA treatment. (f) Fluorescence images of MOC1‐Luc cells after different treatments. (g) Cytotoxicity analysis of ZIF‐8/2‐DG@Cu‐TA against MOC1, HSC3, Cal‐27, and SCC‐25 cells. Data are expressed as means ± SD (*n* = 3). Statistical significance was calculated by one‐way and two‐way ANOVA with Tukey's post hoc test. ^*^ *p* < 0.05, ^**^ *p* < 0.01, ^***^ *p* < 0.001, and ^****^ *p* < 0.0001.

Abundant Zn^2^^+^ within mitochondria drives massive reactive oxygen species (ROS) accumulation and subsequently initiates mitochondria‑mediated apoptosis [33]. 2',7'‑Dichlorodihydrofluorescein diacetate (DCFH‑DA) was used as a probe, which generates intense green fluorescence upon reaction with ROS [34]. As observed in Figure 4c, obvious right shifts were detected for all treatment groups, indicative of elevated ROS levels caused by intracellular Zn^2+^ accumulation. Notably, the ZIF‑8/2‑DG group generated higher ROS levels than the ZIF‑8 group, as 2‑DG contributes to modulating intracellular ROS homeostasis. JC‑1 is a ΔΨ_m_ probe that forms red‑fluorescent aggregates in functionally intact mitochondria and shifts to green‑fluorescent monomers following mitochondrial depolarization [35]. Figure 4d displayed dominant red fluorescence within the PBS group, whereas nanomedicine‑treated groups showed intensified green fluorescence, pointing to mitochondrial damage whose magnitude matched the ROS elevation. Flow cytometry further supported these observations once again (Figure S15).

Upon inhibition of mitochondrial function and glycolysis, cells shift their energy metabolism toward LDs utilization. MOC1‑derived LDs were traced via flow cytometry. LDs fluorescence intensity diminished progressively in MOC1 cells while rising within CAFs as co‑culture proceeded (Figure 4e), indicating progressive LDs transfer from MOC1 to CAFs, reaching maximal transfer efficiency at 12 h. At 24 h, LDs levels declined within CAFs, which may be associated with catabolism of internalized LDs. This LDs transfer was abolished following Cx43‐KD (Figure S16). However, the Cx43‑KD MOC1 cells still exhibited LDs consumption, reflecting metabolic compensation under metabolic‑starvation stress. We further validated HOCAF‑mediated LDs hijacking toward Cal‑27 cells in the co‐culture system (Figure S16d). These observations demonstrate that metabolic stress elicited by ZIF‑8/2‑DG@Cu‑TA remodels tumor‑CAF intercellular metabolic communication and disrupts tumor energy availability via Cx43‑mediated LDs shuttling.

To evaluate the antitumor effect of ZIF‑8/2‑DG@Cu‑TA, we prepared MOC1‑Luc monoculture and MOC1‑Luc‑CAF co‑culture systems and monitored tumor‑cell proliferation via bioluminescence imaging. Bioluminescence assays showed that ZIF‑8/2‑DG@Cu‑TA alone suppressed MOC1 cell growth, and CAF‑mediated LDs competition further markedly enhanced its antitumor efficacy (Figure 4f). We next assessed its in vitro antitumor activity across multiple cell lines, including murine MOC1 and human HSC3, Cal‑27, and SCC‑25 (Figure 4g). Corresponding IC_50_ values were 99.89 µg/mL (MOC1), 156.7 µg/mL (HSC3), 124.4 µg/mL (Cal‑27), and 132.8 µg/mL (SCC‑25) (Figure S17). Synergistic cytotoxicity was detected at 50–100 µg/mL (CI < 1); detailed Bliss and HSA calculations are provided in Figure S18 and supplementary notes. Live‑dead double staining was used to characterize nanomedicine‑induced cell death, which distinguishes membrane‑compromised dead cells from viable cells. Fluorescence imaging displayed the strongest red signal but the weakest green signal in ZIF‑8/2‑DG@Cu‑TA group (Figure S19). Consistent with the above findings, Annexin V staining recorded an elevation in apoptotic cell fraction from 5.59% (PBS) to 38.2% (ZIF‑8/2‑DG@Cu‑TA), further validating potent apoptotic induction (Figure S19). We further evaluated the biological safety of ZIF‑8/2‑DG@Cu‑TA. In hemolysis assays, only 8.4% hemolysis was observed even at the maximum tested concentration of 600 µg/mL (Figure S20), indicating favorable hemocompatibility of the nanosystem. HaCaT and HOMF were treated with ZIF‑8/2‑DG@Cu‑TA for 12 h and 24 h. Cell viability of both cells remained above 85% at 600 µg/mL. These observations indicate favorable biocompatibility of ZIF‑8/2‑DG@Cu‑TA and support further translational studies.

Tumor metabolic collapse is often accompanied by immunogenic cell death. We therefore examined whether ZIF‑8/2‑DG@Cu‑TA could activate antigen‑presenting cells (dendritic cells, DCs). Flow cytometric analysis revealed that the mature DCs reached 37.5% in the ZIF‑8/2‑DG@Cu‑TA group, markedly higher than that in the PBS (11.1%), ZIF‑8 (18.6%), and ZIF‑8/2‑DG (26.2%) groups, and comparable to that in the ZIF‑8@Cu‑TA group (21.9%, Figure S21). These findings suggest that ZIF‑8/2‑DG@Cu‑TA efficiently promotes DCs maturation and holds potential for activating antitumor immunity. This work redefines the role of CAFs in tumor metabolic adaptation. CAFs no longer serve merely as supportive cells for tumor growth. Under nanomedicine‑induced metabolic stress, CAFs undergo metabolic reprogramming and act as tumor‑specific “energy sinks” through Cx43 gap junctions, which indirectly facilitates the initiation of antitumor immune responses.

### 2.5 Mechanism of ZIF‐8/2‐DG@Cu‐TA‐Mediated Metabolic Collapse and Lipid Hijacking via RNA‐seq

We then performed high‑throughput RNA sequencing and transcriptomic profiling of treated MOC1 cells to explore the possible mechanisms. UpSet and Venn analyses identified 10,833 co‑expressed genes between the treatment and control groups, with 6,123 and 224 genes uniquely expressed in each group, respectively (Figure 5a). We identified 9,116 differentially expressed genes (DEGs), among which 5,091 were significantly upregulated and 4,025 were downregulated (Figure 5b). Twenty genes of interest (GOIs) related to energy metabolism, programmed cell death, and immune response were selected for heatmap visualization (Figure 5c). Gene Ontology (GO) enrichment revealed that these GOIs were primarily associated with oxidative phosphorylation, glycolysis, lipid metabolism, and cell‑death processes (Figure 5d), and Kyoto Encyclopedia of Genes and Genomes (KEGG) analysis yielded consistent results (Figure 5e). Notably, both GO and KEGG enrichment highlighted cell‑junction and cell‑adhesion pathways (Figure 5f), suggesting the potential involvement of gap junctions in metabolic reprogramming, consistent with the Cx43‑dependent LDs transfer observed earlier.

**FIGURE 5:**
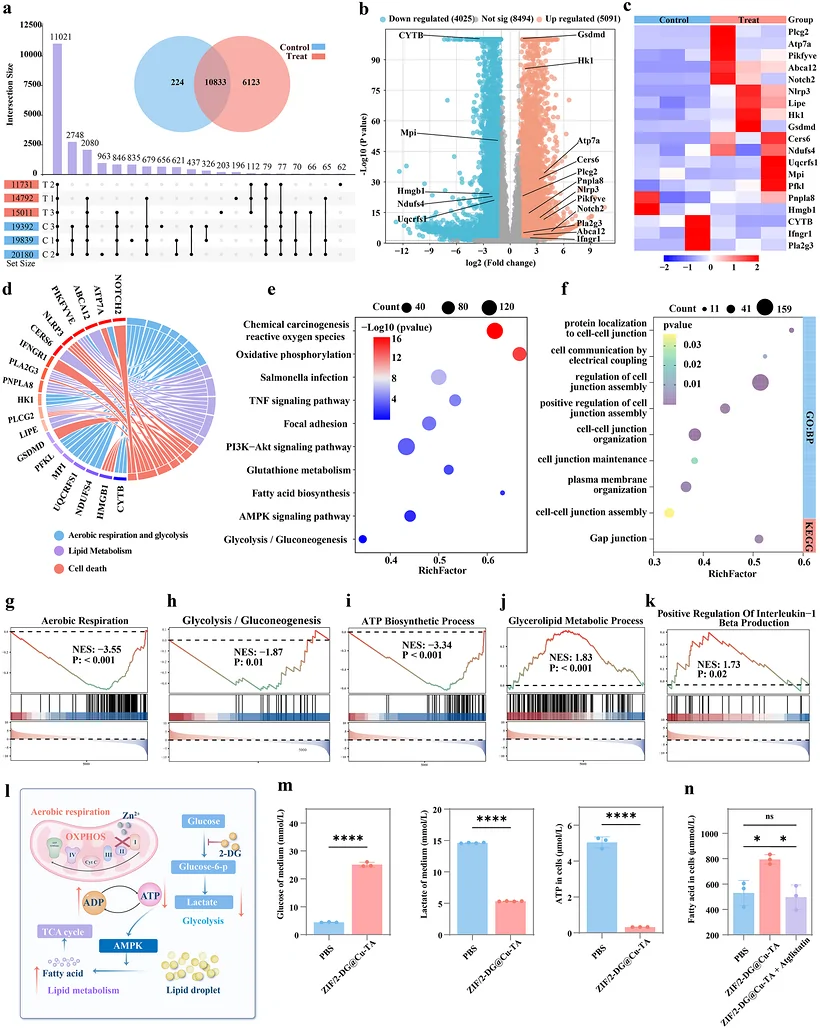
Transcriptomic dissection of the antitumor mechanism of ZIF‐8/2‐DG@Cu‐TA. (a) UpSet plot illustrating gene count distribution and overlap between samples, alongside a Venn diagram. (b) Volcano plot depicting DEGs, highlighting upregulated and downregulated genes. Statistical significance was assessed by two‐sided *t*‐test. (c) Heatmap showing differential expression of GOIs. (d) Circular plot summarizing GO enrichment analysis for GOIs. Statistical significance is assessed by one‐sided *t*‐test. (e) Bubble chart presenting KEGG enrichment analysis results for DEGs. (f) Bubble charts of pathways related to gap junctions in GO and KEGG analyses (z‐score>0). GSEA enrichment plots for (g) aerobic respiration, (h) glycolysis, (i) ATP biosynthetic process, (j) glycerolipid metabolism, and (k) IL‐1β production. Statistical significance was assessed by permutation‐based test with FDR‐adjusted significance (*P*adj < 0.05) using clusterProfiler (v4.19.4.008). (l) Schematic diagram of ZIF‐8/2‐DG@Cu‐TA inducing metabolic reprogramming in MOC1 cells. (m) Glucose levels in culture medium, intracellular lactate, and ATP contents following ZIF‐8/2‐DG@Cu‐TA treatment. Statistical significance was calculated by Student's *t*‐test. ^****^ *p* < 0.0001. (n) Intracellular fatty acid levels after treatments. Statistical significance was calculated by one‐way ANOVA test. ^*^ *p* < 0.05 and *p* > 0.05 p > 0.05 indicates no significant difference. Data are expressed as means ± SD (*n* = 3).

Gene Set Enrichment Analysis (GSEA) further uncovered systematic metabolic reprogramming in tumor cells. Two core energy‑generating pathways, aerobic respiration (NES  =  −3.55) and glycolysis (NES  =  −1.87), were markedly suppressed (Figure 5g,h and Figure S22), accompanied by downregulation of ATP‑biosynthesis gene sets (Figure 5i and Figure S23). Under energy deficit, the AMPK pathway was activated (NES  =  1.45) (Figure S24), as a central cellular energy sensor that triggers compensatory metabolic responses upon ATP depletion [36]. In parallel, lipid‑metabolism pathways were upregulated, with glycerophospholipid metabolism showing the most pronounced enrichment (NES  =  1.83 and 1.84, respectively) (Figure 5j and Figure S25). Given that lipids constitute the core component of LDs, this finding suggests that tumor cells mobilize intracellular LDs to compensate for the energy gap when glycolysis and mitochondrial respiration are blocked. GSEA also revealed upregulation of programmed‑cell‑death pathways, supported by elevated inflammatory‑cytokine gene sets (Figure 5k). Taken together, these transcriptomic data indicate that ZIF‑8/2‑DG@Cu‑TA simultaneously suppresses glycolysis and aerobic respiration in tumor cells, which respond by upregulating lipid metabolism and lipid‑droplet mobilization through metabolic reprogramming to counteract the energy crisis (Figure 5l).

To independently validate the transcriptomic predictions, we measured glucose consumption in the culture medium, intracellular lactate production, and ATP levels. All three parameters were significantly reduced in the treatment group (Figure 5m), consistent with the suppressed glycolysis and oxidative phosphorylation observed at the transcriptional level. Concurrently, intracellular levels of fatty acids and other key lipid metabolites increased markedly. Adipose triglyceride lipase (ATGL) is the core enzyme mediating LDs lipolysis. Treatment with Atglistatin, a specific ATGL inhibitor, significantly reversed the fatty‑acid elevation, confirming that tumor cells indeed rely on ATGL‑driven lipid‑droplet mobilization to release fatty acids for energy supply after metabolic reprogramming (Figure 5n). These results elucidate at both molecular and biochemical levels that tumor cells actively initiate an LDs‑mobilization program under ZIF‐8/2‐DG@Cu‐TA nanomedicine‑imposed stress, which provides the necessary molecular basis for Cx43‑dependent LDs hijacking between CAFs and MOC1 cells.

### 2.6 The Location of LDs With ZIF‐8/2‐DG@Cu‐TA Treatment In Vivo

ZIF‑8/DiR@Cu‑TA was fabricated using near‑infrared DiR dye as a model tracer, with free DiR serving as control to evaluate the pharmacokinetic profile and tumor‑targeting performance of the nanomedicine. In vivo imaging revealed markedly stronger tumor‑associated fluorescence in the ZIF‑8/DiR@Cu‑TA group across all monitored time points, with maximum tumor accumulation achieved at 48 h post‑injection (Figure 6a–c). Ex‑vivo organ imaging at 48 h post‑injection further confirmed its targeting specificity. Free DiR predominantly accumulated in the liver, whereas tumor fluorescence intensity in the ZIF‑8/DiR@Cu‑TA group was approximately 4.3‑fold higher (Figure 6d) [37]. Flow cytometric analysis of tumor tissues showed a 75.2% elevation in DiR‑positive cells for ZIF‑8/DiR@Cu‑TA relative to free DiR (Figure 6e and Figure S26). Pharmacokinetic measurements returned a DiR half‑life (t_1/2_) of ∼1.09 h for ZIF‑8/DiR@Cu‑TA (Figure S27), supporting a prolonged circulation time for robust tumor accumulation.

**FIGURE 6:**
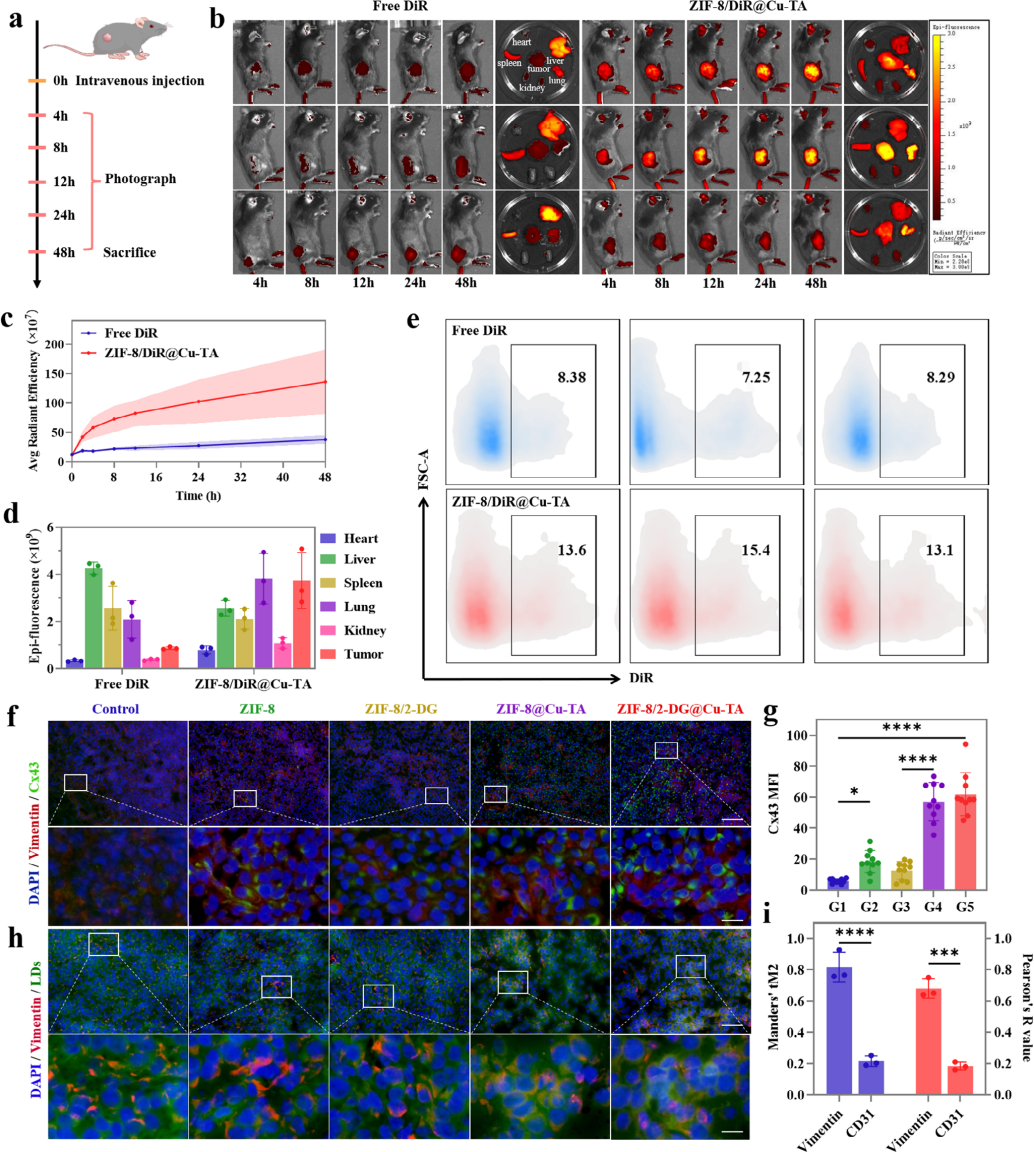
LDs distribution in vivo after ZIF‐8/2‐DG@Cu‐TA treatment. (a) Schematic diagram of the procedure. (b) Fluorescence images of mice and ex vivo harvested tumors, heart, liver, spleen, lungs, and kidneys at 48 h post‐injection. (c) Average fluorescence intensity within tumor regions. (d) Average fluorescence intensity within major organs (heart, liver, spleen, lungs, kidneys). Data are expressed as means ± SD (*n* = 3 mice per group). (e) Proportion of DiR^+^ cells within tumor tissues. (f) Immunofluorescence co‐staining images of vimentin and Cx43 in tumor tissues (scale bar: 100 and 15 µm). (g) Quantification of Cx43 immunofluorescence intensity in tumor tissues. Data are expressed as means ± SD (*n* = 10). (h) Immunofluorescence staining of vimentin and LDs in tumor tissues (scale bar: 50 and 10 µm). (i) Manders' colocalization coefficient (tM2) and colocalization analysis of LDs with vimentin and CD31 in tumor tissues after ZIF‐8/2‐DG@Cu‐TA treatment. Data are expressed as means ± SD (*n* = 3). Statistical significance was calculated by one‐way ANOVA with Tukey's post hoc test. ^*^ *p* < 0.05, ^**^ *p* < 0.001, and ^****^ *p* < 0.0001.

We next investigated LDs redistribution in vivo. Tumor tissues were harvested for analysis 4 days after treatment. Immunofluorescence revealed markedly reduced α‑SMA expression in the ZIF‑8@Cu‑TA and ZIF‑8/2‑DG@Cu‑TA groups, and this observation suggests that the Cu‑TA component may alter the CAF phenotype (Figure S28a). To avoid interference from fluctuating α‑SMA levels in CAF identification, we adopted vimentin, whose expression is relatively stable, as the CAF marker. Immunofluorescence analyses presented in Figure 6f,g confirmed that both ZIF‑8@Cu‑TA and ZIF‑8/2‑DG@Cu‑TA elevate Cx43 expression among CAFs residing inside tumor tissue. Given that Cx43 is also reported to be expressed by endothelial and immune cells, we performed immunofluorescence co‑staining of Cx43 with the endothelial marker CD31 and the immune‑cell marker CD45 (Figure S28). Figure S29 showed high Cx43‑CAF colocalization in oral cancer tissue (Pearson correlation coefficient, PCC  =  0.44); colocalization was weak for endothelial cells (PCC  =  0.11) and nearly absent for immune cells (PCC ≈ 0). These in vivo observations further indicate that CAFs act as the primary effector cells for Cx43‑dependent LDs hijacking. Then, we validated the LDs‑hijacking capacity of CAFs in vivo. Co‑staining of vimentin (red fluorescence) and LDs (green fluorescence) showed that, compared with other treatment groups, green LDs signals prominently clustered around vimentin^+^ CAFs in the ZIF‑8@Cu‑TA and ZIF‑8/2‑DG@Cu‑TA groups (Figure 6h). We further applied the Costes automatic thresholding method combined with Manders' colocalization coefficient (tM2) to quantify the overlap between LDs and the CAF marker or the endothelial marker CD31. Figure 6i and Figure S28e demonstrated that, in the ZIF‑8/2‑DG@Cu‑TA group, the tM2 of vimentin and LDs reached 0.82, whereas that of CD31 and LDs was only 0.21, confirming that LDs^+^ cells within tumor tissue are predominantly CAFs. Colocalization analysis also showed that LDs accumulation in CAFs (PCC  =  0.68) was significantly higher than in endothelial cells (PCC  =  0.18), further confirming CAFs as the major effector cells mediating LDs hijacking. This observed lipid‑redistribution phenotype may reshape material exchange within the tumor microenvironment and potentially exert indirect interference on tumor cell metabolic programmes.

### 2.7 Antitumor Effect of ZIF‐8/2‐DG@Cu‐TA

A subcutaneous MOC1 tumor‐bearing mouse model was used to evaluate the antitumor efficacy of ZIF‑8/2‑DG@Cu‑TA (Figure 7a). On day 14 post‑treatment, the PBS group exhibited aggressive tumor growth with an average volume of approximately 943.65 mm^3^, confirming the high malignancy of MOC1 tumors (Figure 7b). The ZIF‑8 group, which only induced mitochondrial dysfunction, achieved moderate tumor growth inhibition with an average tumor volume of 473.76 mm^3^. Although ZIF‑8/2‑DG disrupted both mitochondrial function and glycolysis, its average tumor volume remained at 459.4 mm^3^ with only limited improvement in antitumor efficacy. This observation may result from compensatory metabolic cascades‑including lipid metabolism‑hijacked by tumor cells to preserve sufficient energy supply. Notably, the ZIF‑8/2‑DG@Cu‑TA group exerted superior tumor suppressive effects, with an average tumor volume of 301.28 mm^3^, merely 31.9% of that measured in the PBS group (Figure 7c). This favorable therapeutic outcome can be ascribed to multi‑faceted interference against three core metabolic pathways, such as mitochondrial function, glycolysis, and lipid metabolism. Tumor weight statistics further corroborated this therapeutic difference; the average tumor weight of the ZIF‑8/2‑DG@Cu‑TA group reached 0.51 g, accounting for only 24% of the 2.09 g in the PBS group (Figure 7d). Representative tumor photographs also exhibited consistent therapeutic trends (Figure 7e). All groups exhibited consistent body weight increases, indicating negligible systemic toxicity (Figure 7f).

**FIGURE 7:**
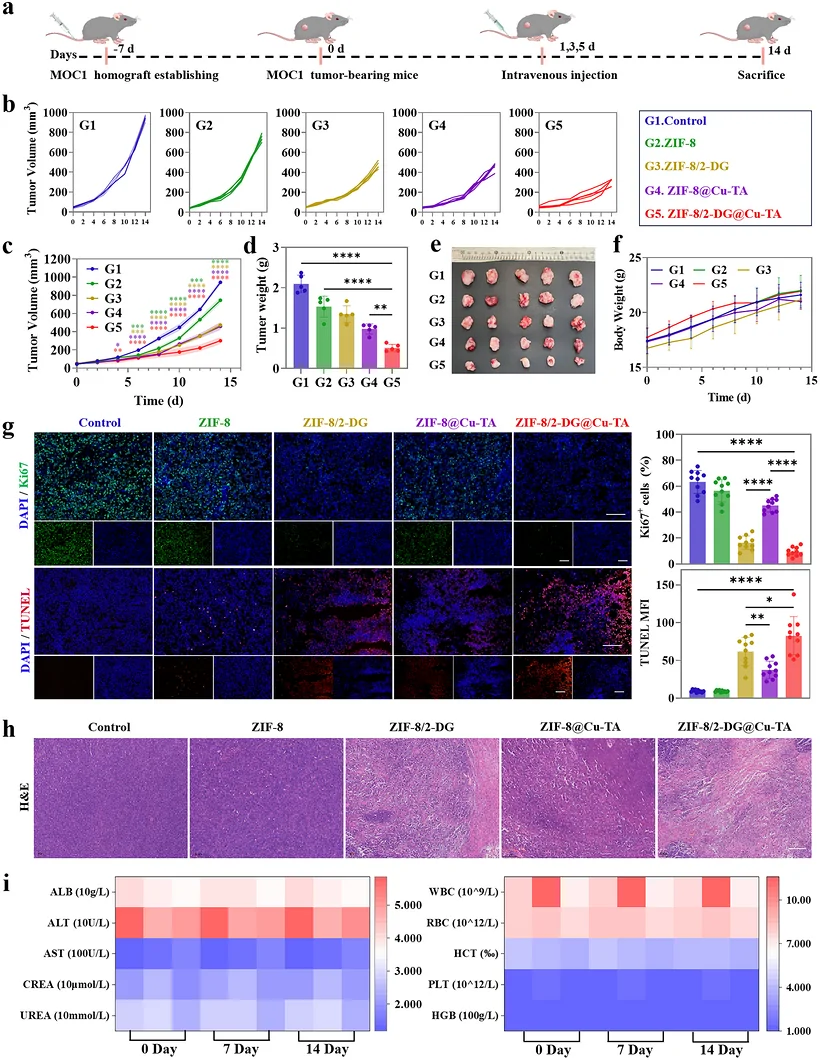
Antitumor efficacy of ZIF‐8/2‐DG@Cu‐TA. (a) Schematic diagram of the therapeutic strategy in MOC1 subcutaneous tumor‐bearing mouse model. (b) Individual tumor growth curves. (c) Average tumor growth curves. (d) Tumor weight. (e) Representative tumor photographs. (f) Body weight. Data are expressed as means ± SD (*n* = 5 mice per group). (g) Immunofluorescence images of Ki67 and TUNEL staining within tumor tissues (scale bar: 100 µm). Data are expressed as means ± SD (*n* = 10). (h) H&E images of tumor tissues. (i) Hematological analysis of mice (*n* = 3) at different time points. Statistical significance was calculated by one‐way/two‐way ANOVA with Tukey's post hoc test. ^*^ *p* < 0.05, ^**^ *p* < 0.01, and ^****^ *p* < 0.0001.

Ki67 and Terminal deoxynucleotidyl transferase‑mediated dUTP nick‑end labelling (TUNEL) immunofluorescence staining is presented in Figure 7g. Tumor tissues from the PBS group displayed strong Ki67 fluorescence but faint TUNEL signals. Upon therapeutic treatment, Ki67 fluorescence gradually declined while TUNEL apoptotic signals increased correspondingly. The ZIF‑8/2‑DG@Cu‑TA group showed the weakest Ki67 intensity and the most prominent TUNEL signals. Quantitative analysis further verified that this group possessed the lowest fraction of proliferation‑positive cells and the highest proportion of apoptotic cells, demonstrating robust suppression of tumor proliferation and markedly elevated apoptotic levels. Hematoxylin‑eosin (H&E) histological staining revealed extensive cellular necrosis and karyopyknosis within tumor regions of the ZIF‑8/2‑DG@Cu‑TA group, further supporting its potent tumor‑killing capacity (Figure 7h). Collectively, these data suggest that multi‑pronged metabolic intervention targeting mitochondria, glycolysis, and lipid metabolism can effectively disable tumor‑cell metabolic compensation, thereby achieving potent in vivo antitumor activity.

To further assess the biosafety profile of this nanomedicine, we performed major‑organ pathological examination and haematological‑biochemical analyses [38]. H&E staining of major organs (heart, liver, spleen, lung, kidney) showed uniform staining and well‑organized cellular architecture, without obvious pathological lesions (Figure S30). We further assessed liver and kidney function in healthy mice following ZIF‑8/2‑DG@Cu‑TA administration (Figure 7i). Routine blood tests and hepatic‑renal function analyses were performed on days 0,  7, and 14 post‑injection. No significant shifts in hematological parameters or signs of hepatic‑renal impairment were observed. This favorable biosafety profile may stem from the intrinsic biocompatibility of ZIF‑8‑based materials as well as EPR‑driven tumor‑targeted delivery, which helps limit non‑specific drug accumulation and associated adverse responses.

### 2.8 In Vivo Immune Response and Orthotopic Tumor Suppression

We further explored the tumor immune microenvironment remodeling capacity of ZIF‑8/2‑DG@Cu‑TA. Building upon the occurrence of immunogenic cell death, effective DC maturation and antigen‑processing function are indispensable to ignite adaptive antitumor immunity. Our results revealed that ZIF‑8/2‑DG@Cu‑TA treatment effectively elevated and sustained DCs maturation and antigen presentation activity (Figure 8a,b and Figures S31 and S36), thereby providing a prerequisite for the activation of systemic antitumor immune responses. Tumor‐infiltrating T cells are pivotal mediators of antitumor immune defense. *t*‑SNE clustering analysis of T cell subsets in tumor tissues showed that all treatment groups exhibited increased infiltration of CD4^+^ and CD8^+^ T cells, with the most prominent improvement observed in the ZIF‑8/2‑DG@Cu‑TA group (Figure 8c). Flow cytometry quantification confirmed that the proportion of cytotoxic CD8^+^ T cells reached 58.9% in the ZIF‑8/2‑DG@Cu‑TA group, 2.76‐fold higher than that in the PBS group (Figure 8d and Figures S32a and S35). Meanwhile, the percentage of CD4^+^ helper T cells increased to 4.6%, which was 3.28‐fold higher relative to the PBS group (Figure 8e). The robust expansion of CD4^+^ T cells was presumably attributed to enhanced antigen recognition and activation induced by mature DCs via MHC‑II peptide complexes [39]. Moreover, ZIF‑8/2‑DG@Cu‑TA treatment dramatically increased the proportion of IFN‑γ‑expressing functional CD8^+^ T cells to 82.73%, which was far superior to the 2.08% recorded in the control group (Figure 8f and Figures S32b and S37). As a critical immunomodulatory cytokine, IFN‑γ strengthens the cytotoxicity of cytotoxic T lymphocytes (CTLs) against tumor cells and facilitates the formation of durable antitumor immune responses [40].

**FIGURE 8:**
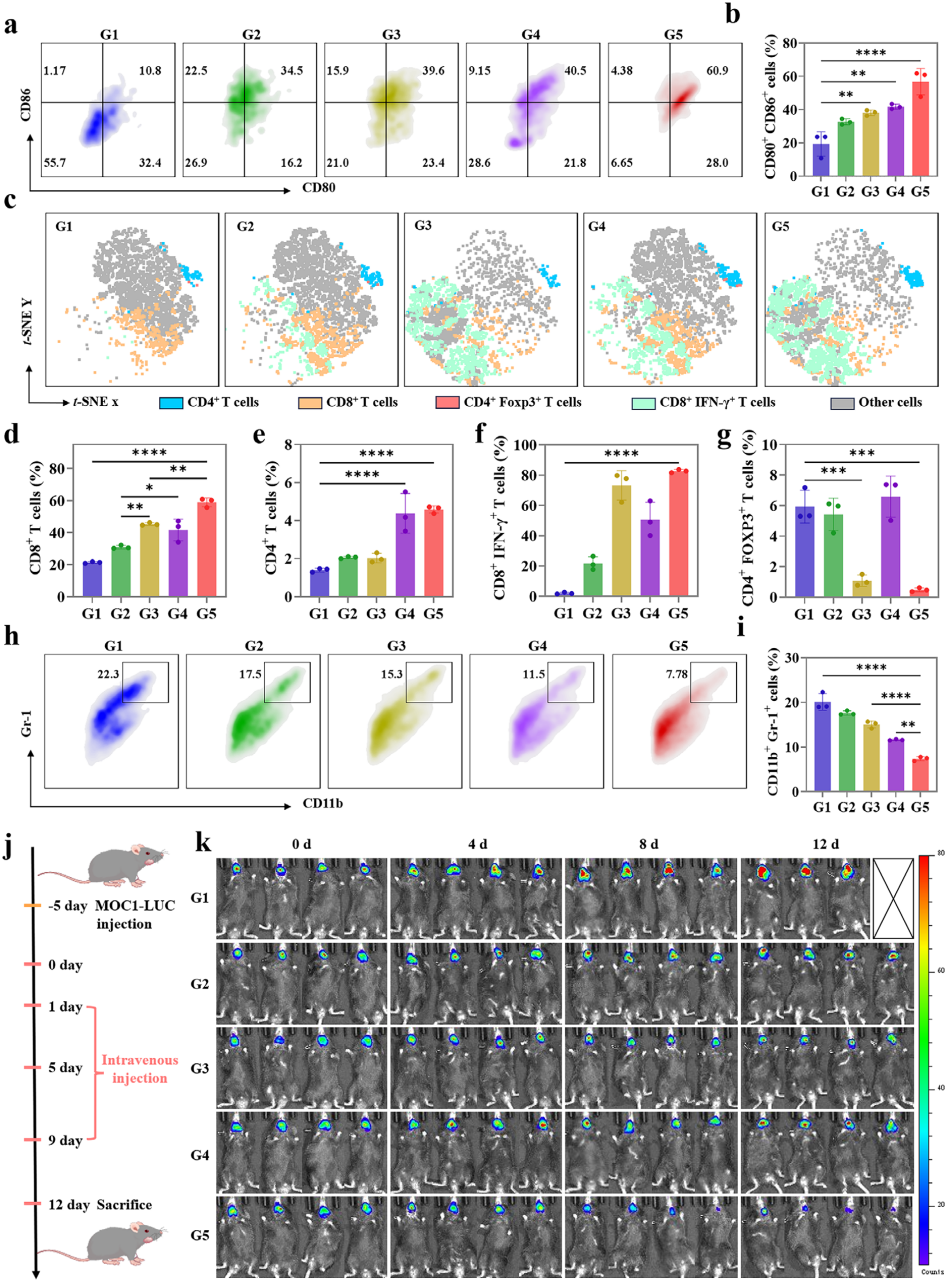
Antitumor immunity and orthotopic therapy of ZIF‐8/2‐DG@Cu‐TA. (a, b) Flow cytometric analysis of mature DCs in tumor‐draining lymph nodes after different treatments (G1: control, G2: ZIF‐8, G3: ZIF‐8/2‐DG, G4: ZIF‐8@Cu‐TA, G5: ZIF‐8/2‐DG@Cu‐TA). (c) *t*‐SNE analysis of tumor‐infiltrating T cells. (d–g) Flow cytometric analysis of CD8^+,^ CD4^+,^ CD8^+^IFN‐γ^+^ and CD4^+^Foxp3^+^ T cells after different treatments. (h, i) Flow cytometric analysis of MDSCs after different treatments. Data are expressed as means ± SD (*n* = 3). (j) Schematic of the MOC1‐LUC orthotopic tumor bearing mouse model and the therapeutic strategy. (k) *B*ioluminescence imaging of mice at different time points after treatment. Statistical significance was calculated by one‐way ANOVA with Tukey's post hoc test. ^*^ *p* < 0.05, ^**^ *p* < 0.01, ^***^ *p* < 0.001, and ^****^ *p* < 0.0001.

Regulatory T cells (Tregs, Foxp3^+^CD4^+^) are key immunosuppressive populations that mediate tumor immune tolerance and immune escape. Notably, the proportion of Tregs was significantly reduced to only 0.5% after ZIF‑8/2‑DG@Cu‑TA treatment (Figure 8g and Figure S32c). The reduction of Tregs efficiently alleviates Foxp3‑driven immunosuppression in the tumor microenvironment, relieves local immune suppression, and creates a favorable immune microenvironment for antitumor immune activation [41]. Myeloid‑derived suppressor cells (MDSCs) and M2‑type tumor‑associated macrophages are major contributors to immunosuppressive tumor microenvironments and tumor progression. In this study, ZIF‑8/2‑DG@Cu‑TA treatment markedly reduced the infiltration of CD11b^+^Gr‑1^+^ MDSCs and M2‑type macrophages, while significantly upregulating the proportion of antitumor M1‑type macrophages to 62.7% (Figure 8h,i and Figure S33). It is worth noting that macrophage polarization represents a dynamic and continuous process rather than a binary switch. The CD80 and CD206 markers applied in this study only reflect the polarization tendency of macrophages, instead of defining absolute M1 or M2 phenotypes. Even so, the obvious phenotypic transformation from M2 to M1 indicates that ZIF‑8/2‑DG@Cu‑TA can effectively remodel the immunosuppressive tumor microenvironment, improve local antigen presentation efficiency, and amplify systemic antitumor immune responses [42]. In summary, ZIF‑8/2‑DG@Cu‑TA realizes efficient and sustained antitumor immune activation through multi‐dimensional modulation of the tumor immune microenvironment.

To better recapitulate the oral cancer microenvironment, we established an MOC1‐LUC orthotopic tumor bearing mouse model, and monitored tumor growth by small‐animal in vivo imaging (Figure 8j). Experimental results showed that mice in PBS group began to die on day 12. To guarantee data integrity and inter‑group comparability across all experimental groups while complying with animal ethics guidelines, day 12 was set as the observational endpoint. As shown in Figure 8k and Figure S34, tumor fluorescence signals in PBS group (G1) continuously increased throughout the 12‑day observation. This observation is consistent with the highly aggressive and lethal features of MOC1 cells and validates that this model well recapitulates the rapid malignant progression observed in clinical OSCC patients. Tumor fluorescence signals in ZIF‑8 (G2), ZIF‑8/2‑DG (G3) and ZIF‑8@Cu‑TA (G4) groups remained stable during the treatment period but exhibited upward trends, which recapitulated the findings obtained from subcutaneous tumor models. In sharp contrast, ZIF‑8/2‑DG@Cu‑TA‑treated group (G5) displayed a distinct tumor‑growth kinetic profile. Tumor fluorescence signals remained largely stable and even decreased to some extent over the entire observation period, with the lowest absolute signal value and nearly negligible net tumor growth. These results indicate that G5 treatment is capable of initiating tumor‑cell cycle arrest or apoptosis shortly after treatment commencement. Such effects sustain low tumor‑burden status and confer long‑lasting tumor inhibition. Moreover, no deaths were recorded among tumor‑bearing mice in group G5 throughout the experiment, whereas mortality events had occurred in the PBS control group by the experimental endpoint. This survival discrepancy suggests that the present nanoplatform may improve the general status of tumor‑bearing mice while potently restraining orthotopic tumor growth. Definitive survival‑benefit assessment nevertheless requires independent experiments with prolonged observation periods. These results provide critical in‑vivo proof‑of‑concept evidence supporting the potential translational value of this nanosystem for metabolic‑intervention‑based OSCC therapy.

## 3 Conclusion

This study identifies LDs trafficking as an actionable vulnerability within tumor metabolic plasticity and establishes intercellular lipid redistribution as a previously underappreciated regulatory layer governing cancer energy homeostasis. We demonstrate that LDs can be directionally redirected from tumor cells into CAFs through Cx43‐mediated gap junctions. This finding unveils a previously unappreciated function of stromal cells within tumor starvation therapy, underscoring that CAFs serve as central regulators of intercellular energy partitioning instead of mere metabolic suppliers. Effective starvation therapy thus requires not only inhibition of core metabolic pathways but also interference with endogenous metabolic compensation. Simultaneous impairment of glycolysis, lipid metabolism and mitochondrial bioenergetics induces irreversible bioenergetic failure, which remodels the tumor immune microenvironment via immunogenic cell death. These observations underscore that overcoming metabolic redundancy demands multilevel, spatially coordinated interventions rather than single‐pathway blockade. Findings based on nanomedicine‐driven rewiring of tumor–stromal metabolic crosstalk are not limited to oral cancer and may prove applicable to other malignancies. Future optimization of intercellular targeting specificity, validation in patient‐derived models and integration with established immunotherapies should further expand the translational potential of this nanoplatform.

## Author Contributions

Conceptualization: X. Zong, H. Yao and Y. Li. Methodology: X. Zong, H. Yao, and Y. Li. Investigation: X. Zong, H. Yao, D. Shen, F. Gong, Z. Wang, H. Qin, and Y. Yang. Visualization: X. Zong, H. Yao, Y. Li, and R. Qiao. Supervision: H. Yao, Y. Li, and R. Li. Writing – original draft: X. Zong, H. Yao, and Y. Li. Writing – review & editing: R. Qiao and R. Li. The authors appreciate the support from Home for Researchers (www.home‐for‐researchers.com) in the preparation of Scheme 1.

## Funding

Y. Li acknowledges the funding support from the National Natural Science Foundation of China (52103190). R. Li acknowledges the funding support from National Natural Science Foundation of China (105732401174), Nature Science Foundation of Henan province (222300420569 and 252300423204), Henan Medical Science and technology research project (SBGJ202002073), Young and Middle‐aged Health Science and Technology Innovation Leading Talents in Henan Province (YXKC2021014), Scientific Research and Innovation Team of the First Affiliated Hospital of Zhengzhou University (QNCXTD2023021), Young and middle‐aged academic leader in Henan Province (HNSWJW‐2021014); and Key project of Medical Science and Technology Research Program of Henan Province (SBGJ202502060). H. Yao acknowledges the funding support from the National Natural Science Foundation of China (32401174) and the Youth Science Fund (Category B) of Jilin Provincial Science and Technology Development Plan (20260101020JJ). R. Q. acknowledges the funding support from the National Health and Medical Research Council and Australian Research Council.

## Conflicts of Interest

The authors declare no conflicts of interest.

## Supporting information




**Supporting File**: advs78459‐sup‐0001‐SuppMat.docx.

## Data Availability

The data that support the findings of this study are available from the corresponding author upon reasonable request.

## References

[1] N. A. Tran , S. S. Moonshi , A. K. Lam , et al., “Nanomaterials in Cancer Starvation Therapy: Pioneering Advances, Therapeutic Potential, And Clinical Challenges,” Cancer and Metastasis Reviews 44, no. 2 (2025): 51, 10.1007/s10555-025-10267-1. PMC12065774 40347350

[2] Y. Zhang , C. Lin , Z. Liu , et al., “Cancer Cells Co‐Opt Nociceptive Nerves To Thrive In Nutrient‐Poor Environments And Upon Nutrient‐Starvation Therapies,” Cell Metabolism 34, no. 12 (2022): 1999–2017.e10, 10.1016/j.cmet.2022.10.012. 36395769

[3] T. Ge , X. Gu , R. Jia , et al., “Crosstalk Between Metabolic Reprogramming And Epigenetics In Cancer: Updates On Mechanisms And Therapeutic Opportunities,” Cancer Communications 42, no. 11 (2022): 1049–1082, 10.1002/cac2.12374. PMC9648395 36266736

[4] Y. Gong , P. Ji , Y. S. Yang , et al., “Metabolic‐Pathway‐Based Subtyping of Triple‐Negative Breast Cancer Reveals Potential Therapeutic Targets,” Cell Metabolism 33, no. 1 (2021): 51–64.e9, 10.1016/j.cmet.2020.10.012. 33181091

[5] M. Martin‐Perez , U. Urdiroz‐Urricelqui , C. Bigas , and S. A. Benitah , “The Role Of Lipids In Cancer Progression And Metastasis,” Cell Metabolism 34, no. 11 (2022): 1675–1699, 10.1016/j.cmet.2022.09.023. 36261043

[6] M. Wan , S. Pan , B. Shan , et al., “Lipid Metabolic Reprograming: The Unsung Hero In Breast Cancer Progression And Tumor Microenvironment,” Molecular Cancer 24, no. 1 (2025): 61, 10.1186/s12943-025-02258-1. PMC11874147 40025508

[7] K. Dm and L. Ca , “Targeting Allosteric Regulation Of Cancer Metabolism,” Nature Chemical Biology 18, no. 5 (2022): 441–450, 10.1038/s41589-022-00997-6. 35484254

[8] Q. Cui , S. Josephraj , B. Gu , J. Y. Liu , and J. T. Zhang , “Targeting Fatty Acid Synthase For Cancer Drug Discovery: Retrospective Analyses And Outlook,” Pharmacological Reviews 78, no. 1 (2026): 100105, 10.1016/j.pharmr.2025.100105. PMC13169002 41519050

[9] C. Mao , D. Jiang , A. C. Koong , and B. Gan , “Exploiting Metabolic Cell Death For Cancer Therapy,” Nature Reviews Cancer 26, no. 1 (2026): 27–45, 10.1038/s41568-025-00879-8. 41094043

[10] S. Goswami , Q. Zhang , C. E. Celik , E. M. Reich , and Ö. H. Yilmaz , “Dietary Fat And Lipid Metabolism In The Tumor Microenvironment,” Biochimica et Biophysica Acta (BBA)—Reviews on Cancer 1878, no. 6 (2023): 188984, 10.1016/j.bbcan.2023.188984. PMC10937091 37722512

[11] H. Xie , X. Liu , Z. Huang , et al., “Nanoscale Zeolitic Imidazolate Framework (ZIF)–8 in Cancer Theranostics: Current Challenges and Prospects,” Cancers 14, no. 16 (2022): 3935, 10.3390/cancers14163935. PMC9405721 36010926

[12] D. Wang , Q. Wu , X. Ren , M. Niu , J. Ren , and X. Meng , “Tunable Zeolitic Imidazolate Framework‐8 Nanoparticles For Biomedical Applications,” Small Methods 8 (2024): 2301270. 10.1002/smtd.202301270 37997211

[13] J. R. Gutierrez‐Camacho , L. Avila‐Carrasco , I. Garza‐Veloz , et al., “Connexin 43 Expression as Biomarker of Oral Squamous Cell Carcinoma and Its Association With Human Papillomavirus 16 and 18,” International Journal of Molecular Sciences 26, no. 3 (2025): 1232, 10.3390/ijms26031232. PMC11818288 39941000

[14] J. Williams , R. Camarda , S. Malkov , et al., “Tumor Cell‐Adipocyte Gap Junctions Activate Lipolysis And Contribute To Breast Tumorigenesis,” Nature Communications 16, no. 1 (2025): 7438, 10.1038/s41467-025-62486-3. PMC12368202 40835606

[15] C. Ni , X. Lou , X. Yao , et al., “ZIP1+ Fibroblasts Protect Lung Cancer Against Chemotherapy Via Connexin‐43 Mediated Intercellular Zn^2+^ Transfer,” Nature Communications 13, no. 1 (2022): 5919, 10.1038/s41467-022-33521-4. PMC9547061 36207295

[16] S. Liao , G. Wu , Z. Xie , et al., “pH Regulators And Their Inhibitors In Tumor Microenvironment,” European Journal of Medicinal Chemistry 267 (2024): 116170, 10.1016/j.ejmech.2024.116170. 38308950

[17] R. Cheng , L. Jiang , H. Gao , et al., “A pH‐Responsive Cluster Metal–Organic Framework Nanoparticle for Enhanced Tumor Accumulation and Antitumor Effect,” Advanced Materials 34, no. 42 (2022): 2203915, 10.1002/adma.202203915. 35985348

[18] H. Jing , X. Huang , X. Du , L. Mo , C. Ma , and H. Wang , “Facile synthesis of pH‐responsive Sodium Alginate/Carboxymethyl Chitosan Hydrogel Beads Promoted By Hydrogen Bond,” Carbohydrate Polymers 278 (2022): 118993, 10.1016/j.carbpol.2021.118993. 34973796

[19] E. Sahai , I. Astsaturov , E. Cukierman , et al., “A Framework For Advancing Our Understanding Of Cancer‐Associated Fibroblasts,” Nature Reviews Cancer 20, no. 3 (2020): 174–186, 10.1038/s41568-019-0238-1. PMC7046529 31980749

[20] C. Guido , D. Whitaker‐Menezes , C. Capparelli , et al., “Metabolic reprogramming of cancer‐associated fibroblasts by TGF‐β drives tumor growth: Connecting TGF‐β signaling With “Warburg‐Like” cancer metabolism and L‐lactate production,” Cell Cycle 11, no. 16 (2012): 3019–3035, 10.4161/cc.21384. PMC3442913 22874531

[21] F. Zhang , Y. Ma , D. Li , et al., “Cancer Associated Fibroblasts And Metabolic Reprogramming: Unraveling The Intricate Crosstalk In Tumor Evolution,” Journal of Hematology & Oncology 17, no. 1 (2024): 80, 10.1186/s13045-024-01600-2. PMC11367794 39223656

[22] G. Q. Zhu , Z. Tang , R. Huang , et al., “CD36+ Cancer‐Associated Fibroblasts Provide Immunosuppressive Microenvironment For Hepatocellular Carcinoma Via Secretion Of Macrophage Migration Inhibitory Factor,” Cell Discovery 9, no. 1 (2023): 25, 10.1038/s41421-023-00529-z. PMC9988869 36878933

[23] L. Héja , Á. Simon , and J. Kardos , “Simulation of Gap Junction Formation Reveals Critical Role Of Cys Disulfide Redox State In Connexin Hemichannel Docking,” Cell Communication and Signaling 22, no. 1 (2024): 185, 10.1186/s12964-023-01439-z. PMC10949817 38500186

[24] C. Hirschhäuser , A. Lissoni , P. M. Görge , et al., “Connexin 43 Phosphorylation By Casein Kinase 1 is Essential For The Cardioprotection By Ischemic Preconditioning,” Basic Research in Cardiology 116, no. 1 (2021): 21, 10.1007/s00395-021-00861-z. PMC7985055 33751227

[25] D. W. Laird and P. D. Lampe , “Cellular Mechanisms Of Connexin‐Based Inherited Diseases,” Trends in Cell Biology 32, no. 1 (2022): 58–69, 10.1016/j.tcb.2021.07.007. PMC8688313 34429228

[26] M. Quan , H. Lv , Z. Liu , et al., “MST1 Suppresses Disturbed Flow Induced Atherosclerosis,” Circulation Research 131, no. 9 (2022): 748–764, 10.1161/CIRCRESAHA.122.321322. 36164986

[27] M. Tufail , C. H. Jiang , and N. Li , “Altered metabolism in cancer: Insights Into Energy Pathways And Therapeutic Targets,” Molecular Cancer 23, no. 1 (2024): 203, 10.1186/s12943-024-02119-3. PMC11409553 39294640

[28] W. Luo , H. Wang , L. Ren , et al., “Adding Fuel To The Fire: The Lipid Droplet And Its Associated Proteins In Cancer Progression,” International Journal of Biological Sciences 18, no. 16 (2022): 6020–6034, 10.7150/ijbs.74902. PMC9682530 36439875

[29] R. P. Norris , “Transfer of Mitochondria And Endosomes Between Cells By Gap Junction Internalization,” Traffic 22, no. 6 (2021): 174–179, 10.1111/tra.12786. 33797162

[30] I. E. García and J. E. Contreras , “Decoding Connexin Hemichannels: Structure, Function, and Regulatory Mechanisms,” Annual Review of Physiology 88, no. 1 (2025): 73–98, 10.1146/annurev-physiol-050724-010008. 41218630

[31] R. Rebelo , C. P. R. Xavier , E. Giovannetti , and M. H. Vasconcelos , “Fibroblasts in pancreatic cancer: Molecular and clinical perspectives,” Trends in Molecular Medicine 29, no. 6 (2023): 439–453, 10.1016/j.molmed.2023.03.002. 37100646

[32] Y. Wang and G. J. Patti , “The Warburg Effect: A Signature Of Mitochondrial Overload,” Trends in Cell Biology 33, no. 12 (2023): 1014–1020, 10.1016/j.tcb.2023.03.013. PMC10600323 37117116

[33] Z. Y. Xi , C. Y. Fan , Y. Y. Jiang , et al., “Nanocatalytic System Releases Overloaded Zinc Ions And ROS To Induce Znproptosis And Interrupt Cell Cycle Through Inhibiting Akt/mTOR pathway,” Theranostics 15, no. 10 (2025): 4734–4762, 10.7150/thno.107025. PMC11984402 40225560

[34] J. Shao , R. Xiao , T. Wang , et al., “BRD4770 protects Against DOX‐induced Cardiotoxicity By Inhibiting Apoptosis And Ferroptosis,” Science Advances 11, no. 28 (2025): adw1720, 10.1126/sciadv.adw1720. PMC12248293 40644536

[35] M. Shao , H. Cheng , X. Li , et al., “Abnormal Mitochondrial Iron Metabolism Damages Alveolar Type II Epithelial Cells Involved In Bleomycin‐Induced Pulmonary Fibrosis,” Theranostics 14 (2024): 2687. 10.7150/thno.94072 PMC11103499 38773980

[36] G. R. Steinberg and D. G. Hardie , “New Insights Into Activation And Function of the AMPK,” Nature Reviews Molecular Cell Biology 24, no. 4 (2023): 255–272, 10.1038/s41580-022-00547-x. 36316383

[37] Z. Cheng , M. Li , R. Dey , and Y. Chen , “Nanomaterials for Cancer Therapy: Current Progress And Perspectives,” Journal of Hematology & Oncology 14, no. 1 (2021): 85, 10.1186/s13045-021-01096-0. PMC8165984 34059100

[38] L. Yang , S. Dong , S. Gai , et al., “Deep Insight of Design, Mechanism, and Cancer Theranostic Strategy of Nanozymes,” Nano‐Micro Letters 16, no. 1 (2023): 28, 10.1007/s40820-023-01224-0. PMC10663430 37989794

[39] X. Yin , S. Chen , and S. C. Eisenbarth , “Dendritic Cell Regulation of T Helper Cells,” Annual Review of Immunology 39, no. 1 (2021): 759–790, 10.1146/annurev-immunol-101819-025146. 33710920

[40] H. Zhang , X. Song , L. Zhao , et al., “Singlet Oxygen‐Precharged Injectable Hydrogel Triggers Ferroptosis And Lactate Metabolism Reprogramming For Enhanced Colorectal Cancer Immunotherapy,” Journal of Nanobiotechnology 24, no. 1 (2026): 186, 10.1186/s12951-026-04054-5. PMC12924412 41606647

[41] X. Chen , M. Ghanizada , V. Mallajosyula , et al., “Differential roles of human CD4^+^ and CD8^+^ regulatory T Cells In Controlling Self‐Reactive Immune Responses,” Nature Immunology 26, no. 2 (2025): 230–239, 10.1038/s41590-024-02062-x. PMC11785521 39806065

[42] Y. S. Lin , H. Y. Chang , W. Z. Hong , et al., “Targeting ENO1 Reprograms Macrophage Polarization To Trigger Antitumor Immunity And Improves The Therapeutic Effect Of Radiotherapy,” Cell Death & Disease 17, no. 1 (2026): 194, 10.1038/s41419-026-08416-7. PMC12876979 41629269

