# Supplementary material for: pH‐Responsive Nanoplatform‐Mediated Lipid Hijacking and Metabolic Collapse for Synergistic Starvation‐Immunotherapy
Source: Adv Sci (Weinh). 2026 Oct 28:e78459. Online ahead of print. doi: 10.1002/advs.78459 (PMC13715918; doi:10.1002/advs.78459)
Supplement: Supplementary file 1 [file ADVS-9999-e78459-s001.docx]

Supporting Information

**pH-Responsive Nanoplatform-Mediated Lipid Hijacking and Metabolic Collapse for Synergistic Starvation-Immunotherapy**

*Xianlong Zong, Haochen Yao^*^, Danyang Shen, Furu Gong, Zixuan Wang, Haoyang Qin, Yang Yang, Ruirui Qiao^*^, Yongjuan Li^*^, Rui Li^*^*

X. Zong, D. Shen, F. Gong, Z. Wang, H. Qin, Y. Yang, Prof. R. Li

Department of Stomatology, The First Affiliated Hospital of Zhengzhou University, Zhengzhou University, Zhengzhou, Henan 450000, China
E-mail: [fcclir@zzu.edu.cn](mailto:fcclir@zzu.edu.cn)

Dr. Y. Li

Academy of Medical Sciences, Tianjian Laboratory of Advanced Biomedical Sciences, Zhengzhou University, Zhengzhou, Henan 450000, China

E-mail: [yjli@zzu.edu.cn](mailto:yjli@zzu.edu.cn)

Prof. H. Yao

Key Laboratory and Engineering Laboratory of Lymphatic Surgery Jilin Province, China-Japan Union Hospital of Jilin University, Changchun, 130033, Jilin, China.

E-mail: [yaohaochen@jlu.edu.cn](mailto:yaohaochen@jlu.edu.cn)

Prof. R. Qiao

Australian Institute for Bioengineering and Nanotechnology, The University of Queensland, QLD 4072, Australia.

E-mail: [r.qiao@uq.edu.au](mailto:r.qiao@uq.edu.au)

**Experimental Section**

*Materials:* Zn(NO₃)₂·6H₂O, 2-methylimidazole, 2-DG, 2-NBDG, CuSO₄·5H₂O, tannic acid (TA), NaOH, [Ru(dpp)₃]Cl₂, and 1,1'-dioctadecyl-3,3,3',3'-tetramethylindocarbocyanine perchlorate (DiI) were purchased from Sigma-Aldrich (Shanghai, China). Rhodamine B hydrazide (RBH, cat. HY-123645), N-(6-methoxy-8-quinolyl)-p-toluenesulfonamide (TSQ, cat. HY-119287), N,N′-bis[4-(4,5-dihydro-1H-imidazol-2-yl)phenyl]-3,3′-p-phenylene-bis-acrylamide dihydrochloride (GW4869, cat. HY-19363), cytochalasin B (Cyto B, cat. HY-16928), 3,3'-dioctadecyloxacarbocyanine perchlorate (DiO, cat. HY-D0969), Block lipid transport-1 (BLT-1, cat. HY-116767), Sulfosuccinimidyl oleate sodium (SSO, cat. HY-112847A) and 1,1'-dioctadecyl-3,3,3',3'-tetramethylindotricarbocyanine iodide (DiR, cat. HY-D1048) were purchased from MedChemExpress (Shanghai, China). BODIPY 493/503 (cat. A411914) was obtained from AmBeed (USA). D-luciferin potassium salt (D812647) was purchased from Macklin (Shanghai, China). DAPI (cat. G1012), Hoechst 33342 (cat. G1127), Cell Counting Kit‑8 (cat. G4103), ATP assay kit (cat. G4309), Calcein AM (cat. G1728), TUNEL Apoptosis Assay Kit (cat. G1516) were purchased from Servicebio (Wuhan, China). Glucose assay kit (cat. A154-1-1), Nonesterified free fatty acids assay kit (cat. A042-2-1), and Lactate dehydrogenase assay kit (cat. A020-2-1) were obtained from Nanjing Jiancheng Bioengineering Institute (Nanjing, China). ROS assay kit (cat. S0033), JC‑1 staining kit (cat. C2003), and Annexin V‑PE apoptosis detection kit (cat. C1065) were purchased from Beyotime (Shanghai, China). Seahorse XF Real-Time ATP Rate Assay Kit (cat. 103592-100), Seahorse XF 1.0 M glucose solution (cat. 103577-100), Seahorse XF 200 mM glutamine solution (cat. 103579-100), and Seahorse XF 100 mM pyruvate solution (cat. 103578-100) were obtained from Agilent (USA). HumanKine® recombinant human TGF-β1 protein (cat. HZ-1011) was purchased from Proteintech (Wuhan, China).

*Synthesis of ZIF-8/2-DG:* 2-Methylimidazole (2.87 g) was dissolved in 10 mL of deionized water (3.5 M). Meanwhile, Zn(NO₃)₂·6H₂O (148.7 mg) and 2-DG (60 mg) were dissolved in 10 mL of deionized water. The Zn(NO₃)₂/2-DG solution was then added dropwise to the 2-methylimidazole solution, and the resulting mixture was stirred for an additional 30 min. The precipitate was collected by centrifugation, washed with deionized water, and vacuum-dried to afford ZIF-8/2-DG.

*Synthesis of ZIF-8/2-DG@Cu-TA:* 875 mg of CuSO₄·5H₂O and 27 mg of TA were dissolved in 50 mL of deionized water. The pH of the solution was adjusted to 7.4 with 2 M NaOH, and the mixture was stirred vigorously at 57 °C for 3 h. The precipitate was collected by centrifugation, washed with deionized water, and dried under vacuum to give Cu-TA. Next, 54 mg of Cu-TA and 100 mg of ZIF-8/2-DG were separately dispersed by ultrasonication in 500 μL of deionized water and 10 mL of 10% ethanol, respectively. The Cu-TA dispersion was then added dropwise to the ZIF-8/2-DG dispersion, and the mixture was stirred for an additional 30 min. Finally, the precipitate was collected by centrifugation, washed with deionized water, and dried under vacuum, affording ZIF-8/2-DG@Cu-TA.

*pH-dependent degradation of ZIF-8@Cu-TA:* 10 mg of ZIF-8@Cu-TA was dispersed in deionized water at pH 6.8 or 5.5. Samples were collected at 4, 8, 12, and 24 h for imaging using TEM.

*Drug release of ZIF-8/2-DG@Cu-TA:* Solutions of ZIF-8/2-DG@Cu-TA at pH 7.4, pH 6.8, and pH 5.5 were prepared and placed in a 37°C incubator. At 1, 2, 4, 8, 12, and 24 h, the samples were centrifuged and 100 µL of the supernatant was collected. The supernatants were then stained with 0.1 mM TSQ and 0.1 mM RBH for 30 min, respectively. The fluorescence intensity was measured using a microplate reader at λex/λem = 344/495 nm for TSQ and λex/λem = 510/578 nm for RBH.

2-DG was first replaced with its fluorescent analog 2-NBDG, and ZIF-8/2-NBDG@Cu-TA was prepared following the same procedure. Solutions of ZIF-8/2-NBDG@Cu-TA at pH 7.4 and pH 5.5 were prepared and placed in a 37°C incubator. At 1, 2, 4, 8, 12, and 24 h, the samples were centrifuged and 100 µL of the supernatant was collected and mixed with 200 µL of hexokinase working solution. The fluorescence intensity was measured using a microplate reader at λex/λem = 485/540 nm.

*Cell culture:* 3T3, HOMF, HaCaT, MOC1, HSC3, SCC-25, and Cal-27 cells were cultured in DMEM medium (Thermo Fisher Scientific), while DC2.4 cells were cultured in RPMI 1640 medium (Thermo Fisher Scientific). And HOCAF cells were cultured in specialized medium for human oral cancer fibroblasts  (Quicell). 3T3-derived CAFs were obtained by subjecting 3T3 cells to TGF‑β1 stimulation at 5 ng/mL for 24 h. All media were supplemented with 10% fetal bovine serum (FBS, cell-box, cat. AUS-01E-02) and 1% penicillin-streptomycin antibiotic mixture (Solarbio, cat. P1400). The cells were maintained in an incubator at 37°C with 5% CO₂.

*Cellular uptake:* For co-culture systems, MOC1 cells were mixed with CAFs labeled with DiO at a 1:1 ratio and seeded in 6-well plates for 24 h. After treatment with PBS, Cu-TA, and ZIF-8/2-DG@Cu-TA for 3 h, the cells were incubated with 10 µM RBH for 2 h. The cells were then washed with PBS and imaged using a fluorescence microscope.

To investigate the mechanism of Cu-TA uptake by CAFs, CAFs were seeded in 6-well plates and cultured for 12 h. And then the medium was replaced with fresh medium containing ZIF-8/2-DG@Cu-TA (100 μg/mL), ZIF-8/2-DG@Cu-TA + sulfo-N-Succinimidyl oleate sodium (SSO, 60 μM), or ZIF-8/2-DG@Cu-TA + Block lipid transport-1 (BLT-1, 60 nM). Following 3 h of incubation, RBH (10 μM) was supplemented for another 2 h. Cells were washed with PBS before imaging using a fluorescence microscope.

For Zn²⁺ detection, MOC1 cells were seeded in 6-well plates and cultured for 12 h. The culture medium was then replaced with fresh medium containing PBS, Zn(NO₃)₂, ZIF-8, ZIF-8/2-DG and ZIF-8/2-DG@Cu-TA, respectively. Following 3 h of incubation, TSQ (20 μM) was supplemented for another 2 h. Cells were washed with PBS before imaging using a fluorescence microscope.

For 2-DG detection, MOC1 cells were first labeled with DiI (1 μM). CAFs were then seeded into the MOC1 culture dishes and co-cultured for 12 h. After removing the original medium, fresh medium containing 2-NBDG, ZIF-8/2-NBDG, or ZIF-8/2-NBDG@Cu-TA (2% FBS) was added. Following 2 h of incubation, cells were washed with PBS and imaged using a fluorescence microscope.

*In vitro cellular characterization:* In the following experiments, unless otherwise specified, MOC1 cells were subjected to identical culture conditions and subsequent treatments. Specifically, MOC1 cells were seeded in 6-well plates and cultured for 12 h. The culture medium was then replaced with fresh medium containing PBS, ZIF-8, ZIF-8/2-DG, ZIF-8 @Cu-TA and ZIF-8/2-DG@Cu-TA respectively, and the cells were cultured for an additional period.

To measure the real-time ATP generation rate of cells, MOC1 cells were seeded at a density of 1000 cells per well in a Seahorse assay plate and cultured for 12 h, while the sensor cartridge was hydrated. Culture medium was then replaced with fresh medium containing respective treatments (PBS, ZIF-8, ZIF-8/2-DG, ZIF-8/2-DG@Cu-TA). Following incubation for an additional 3 or 6 h, cells were washed according to the Seahorse assay protocol, and Oligomycin together with Rotenone/Antimycin A were loaded into the sensor cartridge. The ATP levels were recorded using a Seahorse XF Analyzer.

To detect intracellular ROS, treated MOC1 cells were incubated with DCFH‑DA (10 μM) at 37 °C for 30 min in the dark. Following PBS washing, cells were imaged using a fluorescence microscopy, and corresponding fluorescence intensity was quantified via flow cytometry.

To detect mitochondrial membrane potential, treated MOC1 cells were incubated with JC-1 probe (10 μg/mL) at 37°C for 30 min in the dark. Following PBS washing, cells were imaged using a fluorescence microscopy, and corresponding fluorescence intensity was quantified via flow cytometry.

To assess intercellular molecule transfer, MOC1 cells were first labeled with DiI (1 µM) and then incubated with Calcein-AM (3 µM) in the dark at 37°C for 30 min. CAFs were co-cultured with the DiI-labeled MOC1 cells for 12 h. After stained the co-cultured systems with Hoechst 33342, cells were imaged with a fluorescence microscopy.

To detect intracellular LDs, the treated cells were incubated with BODIPY 493/503 (2 µM) for 30 min. Following stained with Hoechst 33342, cells were imaged with a fluorescence microscope.

To track LDs, CAFs were first stained with the Cell-Tracker Deep Red live-cell tracer (500 nM) for 40 min, washed with PBS, and incubated for another 30 min, while MOC1 and MOC1 Cx43-KD cells were stained with BODIPY 493/503 (2 μM) for 30 min, washed with PBS, and incubated for another 1 h. CAFs were then mixed with MOC1 or MOC1 Cx43-KD cells at a 1:1 ratio in 6-well plates, cultured in fresh medium containing ZIF-8/2-DG@Cu-TA (100 μg/mL), and collected at 0, 4, 8, 12, and 24 h before flow cytometric analysis.

To functionally validate LDs hijacking in HOCAFs, HOCAFs and Cal‑27 cells were used to repeat the above‑mentioned experiments.

To detect apoptosis, MOC1 cells were seeded in 6-well plates and cultured for 12 h. The medium was then replaced with fresh medium containing PBS, ZIF‑8, ZIF‑8/2‑DG, ZIF‑8@Cu‑TA and ZIF‑8/2‑DG@Cu‑TA. Following incubation for another 12 h, Annexin V‑FITC and PI were added following kit instructions. Stained cells were then imaged by fluorescence microscopy, with fluorescence intensity quantified via flow cytometry.

*DCs maturation*: MOC1 cells and CAFs were seeded into 6-well plates at a 1:1 ratio. The medium was then replaced with fresh medium containing PBS, ZIF-8, ZIF-8/2-DG, ZIF-8@Cu-TA, and ZIF-8/2-DG@Cu-TA for 24 h. The supernatant was harvested and mixed with fresh medium at a 1:1 ratio to culture DC2.4 cells for 24 h. Cells were stained for 30 min with anti-CD11c–FITC (117305), anti-CD80–V500 (104741), and anti-CD86–PE (159203). Following PBS washing, cells were analyzed with flow cytometry.

*Functional validation of 3T3-derived CAFs:* 3T3 and CAFs were serum-starved in medium containing 2% FBS for 12 h, then seeded into Transwell inserts. Serum-free medium was added to the upper chamber and medium containing 20% FBS to the lower chamber. Following 12 h incubation, cells were fixed in 4% paraformaldehyde for 20 min and stained with crystal violet for 30 min. Cells remaining on the upper surface of inserts were wiped away, and migrated cells were imaged under a light microscope.

For the wound-healing assay of 3T3-derived CAFs, 3T3 and CAFs were seeded in 6-well plates and cultured for 24 h. Monolayers were scratched, and wound‑closure images were acquired at 0, 12, and 24 h.

*Synergistic effect analysis of ZIF-8/2-DG@Cu-TA*: This study employed the most conservative criterion to assess synergy, namely an HSA excess greater than 0, indicating that the combination had to outperform the best single‑agent group under the condition of equal total dose. In addition, we corrected the Bliss model predictions to eliminate double counting of the ZIF‑8 carrier effect, excluding data points with an inflation error exceeding 10%, and performed two‑way factorial ANOVA as an exploratory test.

Synergistic effect was observed across several cell lines at low concentrations. At 100 µg/mL in SCC-25, Bliss Δ = +12.93% and HSA excess = +20.20% (with ~35% less 2-DG and Cu-TA than the single agents); at this same point CI was borderline additive (0.978)—Bliss assumes independent mechanisms whereas Loewe treats the two agents as dose-equivalent, and because 2-DG and Cu-TA act through distinct pathways, Bliss is the more appropriate reference and is independently confirmed by HSA. At 50 µg/mL in MOC1, CI = 0.786 and HSA excess = +10.49%; at 50 µg/mL in CAL-27, CI = 0.731 and HSA excess = +7.59%. Synergy is thus concentration-specific, robust at 50–100 µg/mL, and not reliably assessable at 200–400 µg/mL where carrier-dose imbalance and ceiling effects dominate; those high-dose points are reported as carrier-limited rather than antagonistic.

*Cx43 knockdown:* MOC1 cells were infected with lentivirus purchased from GenePharma (Suzhou, China). The *Cx43*-shRNA sequence was 5'-TGGTGTCTCTCGCTCTGAATA-3', and a shRNA control lentivirus was used as a negative control. Briefly, MOC1 cells were seeded in 6-well plates. The lentivirus stock was diluted 10-fold with medium and added to the cells (1 mL per well), along with Polybrene (5 μg/mL) for 24 h. After 72 h, cells were subjected to puromycin selection (1.5 μg/mL). The knockdown efficiency was assessed by PCR and Western blot.

In addition MOC1 and MOC1 Cx43-KD cells were seeded in 6-well plates, and then the medium was replaced with DMEM containing 2 μM A23187. Following incubation for another 5 min, the supernatant was collected and measured using an ATP assay kit (Servicebio, G4309).

*Cell Viability Assay*: Cells were seeded in a 96-well plate at a density of 5 × 10³ cells per well and incubated for 24 h. The medium was then replaced with 100 μL of fresh medium containing different concentrations of the nanomedicines, followed by incubation for 12 h or 24 h. Subsequently, 10 μL of CCK-8 was added for 2 h. Absorbance was measured at 450 nm using a microplate reader.

*Immunofluorescence:* Cells were treated according to different experimental conditions. Treated cells were fixed with 4% paraformaldehyde (15 min) and permeabilized with 0.1% Triton X‑100 solution (10 min). After blocking with immunofluorescence blocking solution (30 min Beyotime, China), cells were incubated with primary antibodies (4°C, overnight) and secondary antibodies (37°C, 2 h). Nuclei were stained with DAPI (0.75 μg/mL, 10 min) before imaging via a fluorescence microscope.

*Western blotting:* Cells were treated according to different experimental conditions and placed on ice for lysis in RIPA lysis buffer (Epizyme Biotech, China). Total protein concentrations were detected using a BCA protein assay kit (Solarbio, China). Equal amounts of protein were denatured and separated on 10 % SDS‑polyacrylamide gels, followed by electro‑transfer onto PVDF membranes (Sigma-Aldrich, China). Membranes were blocked with rapid blocking solution (Servicebio, China). Afterwards, membranes were incubated with primary antibodies (4°C overnight) and corresponding secondary antibodies (37°C, 2 h). Finally, protein bands were visualized with a chemiluminescence imaging system (anti‑Cx43 (ab314908, Abcam), anti‑α‑SMA (19245, CST), anti‑Tubulin (2128, CST), anti‑PDGFR α/β (F1540, Selleck), anti‑FAP (ab28244, Abcam).

*RNA-sequencing:* MOC1 cells were divided into a control group and a ZIF-8/2-DG@Cu-TA treated group. Following 6 h of incubation, cells were collected and sent to Pentose Biotechnology Co., Ltd. (Zhengzhou, China) for RNA-seq library construction and sequencing. Briefly, cells were washed with cold PBS. Approximately 1 × 10⁶ cells were lysed directly in TRIzol by repeated pipetting until no visible clumps remained, and the lysate was incubated at room temperature for 5 min. Following centrifugation at 12 000× g for 5 min at 4 °C, the supernatant was transferred to a fresh EP tube containing 0.3 mL of chloroform/isoamyl alcohol (24:1). The mixture was vortexed vigorously for 15 s and centrifuged at 12 000× g for 10 min at 4 °C. The upper aqueous phase containing RNA was transferred to a new tube, mixed with an equal volume of isopropanol, and centrifuged at 12 000× g for 20 min at 4 °C. After discarding the supernatant, the RNA pellet was washed twice with 1 mL of 75% ethanol. Residual ethanol was removed by centrifugation at 12 000× g for 3 min at 4 °C, and the pellet was air‑dried for 5-10 min inside a biosafety cabinet. Finally, 25-100 µL of DEPC‑treated water was added to dissolve the RNA. Total RNA quality and quantity were assessed with a NanoDrop spectrophotometer and an Agilent 2100 bioanalyzer (Thermo Fisher Scientific, MA, USA).

*For mRNA Library Construction:* Oligo(dT)‑coupled magnetic beads were applied for mRNA purification. Purified mRNA was fragmented with fragmentation buffer at an optimized temperature. First‑strand cDNA was synthesized by random hexamer‑primed reverse transcription, followed by second‑strand cDNA synthesis. A‑Tailing Mix and RNA Index Adapters were subsequently added, and the mixture was incubated to perform end repair. The resulting cDNA fragments were amplified via PCR. Amplified products were purified using Ampure XP Beads and eluted in EB solution, and library quality was assessed with an Agilent Technologies 2100 bioanalyzer.

Double‑stranded PCR products were heat‑denatured and circularized using splint oligo sequences to produce single‑stranded circular DNA (ssCir DNA), which served as the final library. The library was amplified by phi29 polymerase to generate DNA nanoballs (DNBs), each harbouring more than 300 copies of an individual molecule. DNBs were loaded onto patterned nanoarrays, and single‑end 50‑base reads were acquired on the BGIseq500 platform (BGI‑Shenzhen, China).

*Biodistribution and pharmacokinetics of ZIF-8/2-DG@Cu-TA:* Mice bearing subcutaneous MOC1 tumors received intravenous administration of DiR or ZIF-8/DiR@Cu-TA (10 mg/kg). Blood was collected from the retro-orbital sinus at 0, 2 min, 4 min, 8 min, 15 min, 30 min, 1 h, 2 h, 4 h, 8 h, 12 h, and 24 h, and then imaged using a small animal *in vivo* imaging system. On the other hand, mice were imaged at 0, 2 h, 4 h, 8 h, 12 h, 24 h, and 48 h. At 48 h postinjection, major organs (heart, liver, spleen, lung, kidney) and tumor were harvested and imaged. Subsequently, the DiR fluorescence intensity in tumor tissues was analyzed using flow cytometry.

*Hemolysis assay*: Blood collected from C57BL/6 mice was repeatedly washed with saline until the supernatant turned clear. The pelleted red blood cells (RBCs) were resuspended in saline to prepare a 2% RBC suspension. Deionized water and saline were used as positive and negative controls, respectively. ZIF‑8/2‑DG@Cu‑TA was serially diluted in saline to obtain suspensions of 50, 100, 200, 300, 400, 500 and 600 μg/mL. Each sample solution was mixed 1:1 (*v/v*) with the 2% RBC suspension, incubated at 37 °C for 3 h, and centrifuged at 3000 rpm for 5 min. The absorbance of supernatants was measured at 540 nm using a microplate reader.

*In vivo therapeutic evaluation of ZIF-8/2-DG@Cu-TA:* Mice bearing MOC1 tumors received intravenous administration of G1‑G5 at 10 mg/kg and were euthanized on day 4. Tumor tissues were collected for immunofluorescence staining of Cx43 and Vimentin (G1: control, G2: ZIF-8, G3: ZIF-8/2-DG, G4: ZIF-8@Cu-TA, G5: ZIF-8/2-DG@Cu-TA).

Tumor‑bearing mice received intravenous administration of G1‑G5 formulations at 10 mg/kg on days 0, 3, and 6. Body weight and tumor volumes were recorded every 2 days. Tumor volume was calculated according to *V=lw^2^/2*. Tumor tissues and major organs were harvested on day 14 for H&E staining, TUNEL assay, and immunofluorescence staining.

*In vivo antitumor immunity:* Tumor-draining lymph nodes were collected, homogenized, and filtered to obtain single-cell suspensions in cold PBS. DAPI (0.75 μg/mL) was added and incubated for 10 min. After PBS washing, Fc receptors were blocked using anti‑mouse CD16/CD32 antibody, and samples were incubated at 4 °C for 10 min. Cells were stained with anti-CD11c-FITC (117305), anti-CD80-V500 (104741) antibody, anti-CD86-PE (159203), anti-CD40-PE (157505) antibody and anti-MHC II-PE (107607) antibody for 30 min before analysis using a flow cytometer.

Tumor tissues were collected, homogenized, and filtered to obtain single-cell suspensions in cold PBS. DAPI (0.75 μg/mL) was added and incubated for 10 min. After PBS washing, Fc receptor were blocked using anti‑mouse CD16/CD32 antibody, and samples were incubated at 4 °C for 10 min. Cells were subsequently stained with corresponding antibodies. After additional PBS washes, proportion of immune cells was assessed by flow cytometry.

For T cells staining: Cells were stained with anti-CD45-BV605 (157217), anti-CD3-FITC (100203), anti-CD4-PE/Cyanine 7 (100421), anti-CD8-PerCP/Cyanine5.5 (155013) for 30 min. After PBS washing, cells were resuspended in medium containing PMA (50 ng/mL), ionomycin (1 μg/mL), and Brefeldin A (10 μg/mL), followed by incubation at 37°C with 5% CO₂ for 5 h. After permeabilization with permeabilized with eBioscience Permeabilization working solution, cells were incubated with anti-Foxp3-PE (118903) and anti-IFN-γ-APC/Cyanine7 (505849) antibodies in permeabilization buffer for 30 min.

For macrophage staining: Cells were stained with anti-CD45-BV605 (157217), anti-CD11b-FITC (101205), anti-F4/80-APC/Cyanine7 (157315), and anti-CD80-V500 (104741) antibody for 30 min. After permeabilization with eBioscience Permeabilization working solution, cells were incubated with anti-CD206-PE/Cyanine7 (141719) antibodies for 30 min.

For MDSCs staining: Cells were stained for 30 min with anti-CD45-BV605 (157217), anti-CD11b-FITC (101205) and anti-GR-1-V500 (108457) antibody.

*Orthotopic therapy of ZIF-8/2-DG@Cu-TA:* MOC1-LUC cells were inoculated between the genioglossus and geniohyoid muscles of C57BL/6 mice to construct an orthotopic oral tumor model. G1-5 groups were used. G1: Control; G2: ZIF-8; G3: ZIF-8/2-DG; G4: ZIF-8@Cu-TA; G5: ZIF-8/2-DG@Cu-TA (*n* = 4). Following intravenous administration at 10 mg/kg on days 1, 5, and 9, D‑Luciferin potassium salt (150 mg/kg) was intraperitoneally injected on days 4, 8, and 12, and bioluminescence signals were recorded with an *in vivo* live imaging system.

*Statistical analysis:* Quantitative data are presented as mean ± SD. Statistical analyses were performed using GraphPad Prism 9.0 and Origin 2021 software. The methods of analysis included Student's *t*-test and one/two-way ANOVA. The criteria for statistical significance were set as: * *P* < 0.05, ** *P* < 0.01, *** *P* < 0.001, **** *P* < 0.0001.

**Figure S1.** XPS spectra of Cu-TA, ZIF-8/2-DG and ZIF-8/2-DG@Cu-TA.

**Figure S2.** High-resolution XPS spectra of Zn 2p for ZIF-8/2-DG@Cu-TA.

**Figure S3.** The ratio of Cu(I) to Cu(II) of ZIF-8/2-DG@Cu-TA in high-resolution XPS spectra.

**Figure S4.** High-resolution XPS spectra of O 1s for ZIF-8/2-DG.

**Figure S5.** Fluorescence spectra of 2-NBDG in ZIF-8/2-NBDG@Cu-TA and ZIF-8@Cu-TA.

**Figure S6.** Zeta potentials of ZIF-8/2-DG, Cu-TA and ZIF-8/2-DG@Cu-TA. Data are expressed as means ± SD (*n* = 3).

**Figure S7.** Tyndall effect of ZIF-8/2-DG@Cu-TA. (a) pH 6.8 and (b) 5.5.

**Figure S8.** CAFs driven from TGF-β-incubated 3T3 cells. (a) Protein expression levels of α-SMA, PDGFR, and FAP. (b) Cell migration ability (scale bar: 50 μm). (c, d) Scratch assay images and quantitative analysis. Data are expressed as means ± SD (*n* = 3). Statistical significance was calculated by one-way ANOVA. ***P* < 0.01.

**Figure S9.** Intracellular Cu^2+^ levels in CAFs. (a) Intracellular Cu^2+^ levels in CAFs after different treatments (CuCl_2_, Cu-TA, ZIF-8@Cu-TA, ZIF-8/2-DG@Cu-TA). (b) Fluorescence images of intracellular Cu²⁺ levels in CAFs after blockade of CD36 and SR-B1 (scale bar: 50 μm).

**Figure S10.** Fluorescence images of 2-NBDG in the MOC1/CAFs co-culture system (scale bar: 50 μm).

**Figure S11.** The expression of Cx43. (a) MFI of Cx43 in CAFs after treatment with ZIF-8/2-DG and ZIF-8/2-DG@Cu-TA. Data are expressed as means ± SD (*n* = 3). (b) Western blot analysis of Cx43 in HOCAF. Statistical significance was calculated by one-way ANOVA. ****P* < 0.001 and *P* > 0.05 is not significant.

**Figure S12.** Fluorescence images of LDs in CAFs after treatment with ZIF-8/2-DG and ZIF-8/2-DG@Cu-TA (scale bar: 100 μm).

**Figure S13.** Validation of Cx43 knockdown efficiency. (a) Western blot analysis of Cx43 in MOC1 cells before and after knockdown. (b) Cx43 gene levels in MOC1 cells before and after Cx43 knockdown using PCR. (c) ATP release through Cx43 in MOC1 cells before and after Cx43 knockdown. Data are expressed as means ± SD (*n* = 3). Statistical significance was calculated by Student's *t*-test and one-way ANOVA with Tukey's post hoc test. *****P* < 0.0001.

**Figure S14.** Detection of ATP production rate in MOC1 cells after different treatments for 3 h. Data are expressed as means ± SD (*n* = 3).

**Figure S15.** Flow cytometry profiles of JC-1 in MOC1 cells after different treatments.

**Figure S16.** Validation of ZIF-8/2-DG@Cu-TA-induced LDs hijacking of MOC1 by CAFs. (a, b) Flow cytometric analysis of LDs fluorescence intensity in the MOC1/CAFs and MOC1 Cx43-KD/CAFs co-culture systems after different treatments. (c) Flow cytometric analysis of the distribution of LDs in MOC1 Cx43-KD cells after ZIF-8/2-DG@Cu-TA treatment within the MOC1 Cx43-KD/CAFs co-culture system. (d) Flow cytometric analysis of the distribution of LDs in Cal-27 cells after ZIF-8/2-DG@Cu-TA treatment within the Cal-27/HOCAF co-culture system. Data are expressed as means ± SD (*n* = 3). Statistical significance was calculated by one-way ANOVA with Tukey's post hoc test. **P* < 0.05, ***P* < 0.01

**Figure S17.** IC_50_ analysis of ZIF-8/2-DG@Cu-TA in MOC1, HSC3, Cal-27, and SCC-25 cells. Data are expressed as means ± SD (*n* = 3).

**Figure S18.** Synergistic effect analysis of ZIF-8/2-DG@Cu-TA. (a) Cell viability of MOC1, Cal-27, HSC3, and SCC-25 cells after different treatments. Data are expressed as means ± SD (*n* = 3). (b) Bliss Delta heatmap (reliable bold, confounded gray). (c) Combination Index heatmap (CI < 0.9 Synergy; CI 0.9–1.1 Additive; CI > 1.1 Antagonism; CI *n.d.* = not determined, because the combination effect fell outside the single-agent range). (d) Bliss Independence bar charts; reliable synergy points are defined as those with Bliss delta > 5%. (e) HSA bar charts. *Combination group exceeding the best single agent (HSA excess > 0)*. (f) Dose–response curves.

**Figure S19.** Fluorescence images and flow cytometry analysis of ZIF-8/2-DG@Cu-TA induced apoptosis in MOC1 cells (scale bar: 50 μm).

**Figure S20.** Biocompatibility of ZIF-8/2-DG@Cu-TA. (a) Images and hemolysis-rate analysis of ZIF-8/2-DG@Cu-TA at different concentrations. (b, c) Cell viability of HaCat and HOMF after treatment with concentrations of ZIF-8/2-DG@Cu-TA. Data are expressed as means ± SD (*n* = 3).

**Figure S21.** Flow cytometric analysis of mature DC2.4 cells. Data are expressed as means ± SD (*n* = 3). Statistical significance was calculated by one-way ANOVA with Tukey's post hoc test. *****P* < 0.0001.

**Figure S22.** Normalized Enrichment Scores (NES) of aerobic respiration and glycolysis-related pathways in MOC1 cell.

**Figure S23.** GSEA analysis of ATP synthesis pathways. Statistical significance is assessed by permutation-based test with FDR-adjusted significance (*P*adj < 0.05) of clusterProfiler (v4.19.4.008).

**Figure S24.** GSEA analysis of AMPK signaling pathway. Statistical significance is assessed by permutation-based test with FDR-adjusted significance (*P*adj < 0.05) of clusterProfiler (v4.19.4.008).

**Figure S25.** NES of lipid metabolism-related pathways in MOC1 cells.

**Figure S26.** Proportion of DiR^+^ cells in tumor tissues analyzed using flow cytometry. Data are expressed as means ± SD (*n* = 3). Statistical significance was calculated by Student's *t*-test. ***P* < 0.01.

**Figure S27.** Pharmacokinetics of ZIF-8/2-DG@Cu-TA in mice. (a) Fluorescence images of ZIF-8/DiR@Cu-TA in blood at different time points. (b) Pharmacokinetic profile of ZIF-8/DiR@Cu-TA in circulation (fitted curve). Data are expressed as means ± SD (*n* = 3).

**Figure S28.** Immunofluorescence staining of tumor tissues. (a) α-SMA (scale bar: 100 μm). (b) CD31 and Cx43 (scale bar: 100 and 15 μm). (c) CD45 and Cx43 (scale bar: 100 and 15 μm). (d) Cx43 (scale bar: 100 and 15 μm). (e) Images and colocalization analysis of vimentin and LDs (scale bar: 50 μm).

**Figure S29.** Colocalization analysis of Cx43 with CD45, CD31, and Vimentin in tumor tissues after ZIF-8/2-DG@Cu-TA treatment. Data are expressed as means ± SD (*n* = 3). Statistical significance was calculated by one-way ANOVA with Tukey's post hoc test. **P* < 0.05, ****P* < 0.001 and *****P* < 0.0001.

**Figure S30.** H&E staining of heart, liver, spleen, lung, and kidney from mice in different treatments (scale bar: 200 μm).

**Figure S31.** Flow cytometry profiles of DCs in tumor-draining lymph nodes after different treatments. (a) CD11c^+^ CD40^+^ cells and (b) CD11c^+^ MHC II^+^ cells. Data are expressed as means ± SD (*n* = 3 mice per group). Statistical significance was calculated by one-way ANOVA with Tukey's post hoc test. *****P* < 0.0001.

**Figure S32.** Flow cytometry profiles of (a) T cells, (b) IFN-γ^+^ CD8^+^ cells and (c) Foxp3^+^ CD4^+^ cells in tumor tissues after different treatments.

**Figure S33.** Flow cytometry profiles of macrophages in tumor tissues after different treatments. Data are expressed as means ± SD (*n* = 3). Statistical significance was calculated by one-way ANOVA with Tukey's post hoc test. *****P* < 0.0001.

**Figure S34.** Antiorthotopic therapy. (a) Quantification of average bioluminescence signals in mice at different time points. Data are expressed as means ± SD (*n* = 4). (b) Quantification of individual bioluminescence signals in mice at different time points.

| T cell | CD8-PerCP-Cy5-5 | CD4-PE-Cy7 |
| --- | --- | --- |
| CD8-PerCP-Cy5-5 | 1 | 0.24 |
| CD4-PE-Cy7 | 0.07 | 1 |

**Figure S35.** Representative gating strategies and compensation settings for T cell flow cytometry channels.

| DCs | CD11c-FITC | MHC II-PE |
| --- | --- | --- |
| CD11c-FITC | 1 | 0 |
| MHC II-PE | 0.5 | 1 |

**Figure S36.** Representative gating strategies for analyzing DCs and compensation settings for DCs flow cytometry channels.

| Macrophage | CD11b-FITC | CD206-PE-Cy7 | CD80-APC | F4/80-APC-Cy7 |
| --- | --- | --- | --- | --- |
| CD11b-FITC | 1 | 0.008817 | 0 | 0.001556 |
| CD206-PE-Cy7 | 0.002604 | 1 | 0.01 | 0.11 |
| CD80-APC | 0.017817 | 0.004454 | 1 | 0.002116 |
| F4/80-APC-Cy7 | 0.027833 | 0.015905 | 0 | 1 |

**Figure S37.** Representative gating strategies and compensation settings for macrophage and MDSC flow cytometry channels.
